# Impact of male partner involvement on mother-to-child transmission of HIV and HIV-free survival among HIV-exposed infants in rural South Africa: Results from a two phase randomised controlled trial

**DOI:** 10.1371/journal.pone.0217467

**Published:** 2019-06-05

**Authors:** Sibusiso Sifunda, Karl Peltzer, Violeta J. Rodriguez, Lissa N. Mandell, Tae Kyoung Lee, Shandir Ramlagan, Maria L. Alcaide, Stephen M. Weiss, Deborah L. Jones

**Affiliations:** 1 Social Aspects of Public Health (SAPH) Research Programme, Human Sciences Research Council, Pretoria, South Africa; 2 Department of Research & Innovation, University of Limpopo, Sovenga, South Africa; 3 Department of Psychiatry & Behavioral Sciences, University of Miami Miller School of Medicine, Miami, FL, United States of America; 4 Department of Psychology, University of Georgia, Athens, GA, United States of America; 5 Ford Foundation Fellow, National Academies of Sciences, Engineering, and Medicine, Washington, DC, United States of America; 6 Department of Public Health Sciences, University of Miami Miller School of Medicine, Miami, FL, United States of America; 7 Department of Medicine, Division of Infectious Diseases, University of Miami Miller School of Medicine, Miami, FL, United States of America; International AIDS Vaccine Initiative, UNITED STATES

## Abstract

**Background:**

The Sub-Saharan Africa region still remains the epicentre of the global HIV/AIDS epidemic. With regards to new paediatric HIV infections, almost 90% of new HIV infections are among children (aged 0–14 years), largely through mother to child transmission. Male Partner Involvement in Prevention of Mother to Child Transmission programmes is now strongly advocated as being key in improving infant outcomes. This study describes the role of Male Partner Involvement on infant HIV infection and mortality survival in the first year among HIV-exposed infants born from HIV positive mothers.

**Methods:**

This study was a two-phase, two condition (intervention or control) longitudinal study as part of a clinic-randomized Prevention of Mother to Child Transmission controlled trial. For Phase 1, female participants were recruited without their male partners. In Phase 2, both female and male participants were enrolled in the study as couples in order to encourage active Male Partner Involvement during pregnancy. Participants had two assessments prenatally (8–24 weeks and 32 weeks) and three assessments postnatally (6 weeks, 6 months, and 12 months)

**Results:**

About 1424 women were eligible for recruitment into the study and 18 eligible women declined to participate. All women had a partner; 54% were unmarried, 26% were cohabiting, and 20% were married. Just over half (55%) of the women had been diagnosed with HIV during the current pregnancy. Phase 1 had significantly more HIV-infected infants than Phase 2 at 12-months postpartum (aOR = 4.55 [1.38, 15.07]). Increased depressive symptoms were associated with infant HIV infection at 12-months (aOR = 1.06 [1.01, 1.10]). Phase 1 also had a significantly greater proportion of dead and HIV-infected infants than Phase 2 at 12-months (aOR = 1.98 [1.33, 2.94]).

**Conclusion:**

Male partner involvement in antenatal care is critical in ensuring infant survival and HIV infection among children born to HIV-positive mothers. This study highlights the high risk of ante-and-post natal depression and underscores the need of screening for depression during pregnancy.

**Trial registration:**

ClinicalTrials.Gov; Trial Number NCT02085356.

## Introduction

The Sub-Saharan Africa (SSA) region still remains the epicentre of the global HIV/AIDS epidemic, with Eastern and Southern Africa being the most affected. Among the 1.8 million new HIV infections in 2017, the SSA region accounted for more than half [1]. Regarding new paediatric HIV infections, SSA bears an even greater burden; in 2017, almost 90% of new HIV infections among children (aged 0–14 years) were in SSA [1]. Over the years, large strides have been made, and rates of new HIV infections have shown a sharp decline among children due to the successful implementation of prevention of mother-to-child transmission (PMTCT) programmes [2]. Despite those successes gained from large-scale PMTCT rollout, about 180,000 children were reported to be newly infected with HIV in 2017 (UNAIDS, 2018), and mother-to-child transmission (MTCT) is largely responsible for most new cases in this age group[1,2]. South Africa (SA) has made tremendous progress with its PMTCT programmes and there has been a reduction in neonatal infections in the last decade. However, rural areas and other resource-scarce settings still face challenges in achieving optimal levels of PMTCT implementation in this country [3].

Earlier PMTCT programmes tended to focus mainly on women and children with little emphasis being placed on male partner involvement (MPI) in antenatal care [4]. MPI in antenatal care as part of a holistic approach in PMTCT programmes has recently been strongly advocated as being key in enhancing and improving infant outcomes [4]. The South African National Strategic Plan for HIV/AIDS care and management places MPI as a key component of a successful PMTCT strategy [5]. Male partners can play an influential role in PMTCT protocol participation in various ways, such as through providing an emotionally supportive atmosphere, encouraging and assisting with adherence to PMTCT practices, and facilitating antenatal clinic attendance by providing transportation or holding the mother’s place in the queue[4,6,7]. Male partner involvement in PMTCT programmes has been reported to lead to higher uptake of PMTCT among women, improved adherence to antiretroviral therapy (ART) and safer infant feeding practices, as well as reducing dropouts from PMTCT programmes[4,8,9], thus reducing MTCT of HIV[4,10,11,12]. In addition, it has been suggested that MPI has a positive impact on the survival of infants born to HIV-positive mothers [13, 14]. Furthermore, MPI in antenatal care provides an opportunity to enhance the uptake of HIV testing and counselling among partners of HIV-positive pregnant women [4].

Even though there has been a recent realisation and enthusiasm towards MPI in maternal health care, the actual rates of male participation in antenatal care among indigenous African communities in SA remain relatively low[15].

There have been few studies reporting on the development and implementation of targeted, contextually relevant behavioural interventions that are aimed at increasing MPI in PMTCT programmes and antenatal care[16,17], and among the limited existing research, most of it focuses on uptake of PMTCT practices (e.g., maternal ART[18] and not on infant health or survival outcomes. While PMTCT practices are key for prevention of vertical transmission, there is a need to examine the role of MPI and the end goal of those practices (i.e., infant HIV-free survival). This study reports on data from a randomised controlled trial that aimed at enhancing MPI in PMTCT programmes in rural communities in South Africa by evaluating its outcomes by intervention status (sessions addressing couples’ issues), phase status (male participation), and the combination of intervention phase. This study describes the role of MPI on infant HIV infection and mortality survival in the first year among HIV-exposed infants born from mothers participating in PMTCT.

## Methods

### Study design

This study was a two-phase, two condition (intervention or control) longitudinal study as part of a clinic-randomised PMTCT controlled trial. Eligible Participants were HIV-infected pregnant women at least 18 years of age, having a male partner, and were recruited between April 10, 2014 and January 30, 2017.

The trial aimed at increasing PMTCT uptake, family planning and male partner participation in the antenatal and postnatal process in 12 randomly selected community health centres in Gert Sibande and Nkangala districts in Mpumalanga province, South Africa [16]. Participants had two assessments prenatally (8–24 weeks and 32 weeks pregnant) and three assessments postnatally (6 weeks, 6 months, and 12 months). For Phase 1, female participants were recruited without their male partners. For Phase 2, both female and male participants were enrolled in the study as couples in order to encourage active MPI during pregnancy and enhance both maternal and infant outcomes.

### Sample and procedures

Eligible participants were HIV-seropositive pregnant women, between 8 and 24 weeks pregnant (typical time of entry into antenatal care), with male partners and aged 18 years or older. In Phase 1, their partners were not enrolled and did not participate in the study. In Phase 2, the women’s partners were also enrolled and participated in study procedures. Those agreeing to participate were enrolled following provision of informed consent.

Subsequent to enrolment, all participants (both men and women) completed a baseline survey in their preferred language (English, isiZulu, or seSotho) using Questionnaire Development System Audio Computer-Assisted Self-Interview (ACASI) software in order to enhance disclosure, accommodate all levels of literacy and reduce interviewer bias. To familiarize participants with the software, assessors completed the demographic component of the questionnaire with participants prior to completion of all other assessments. In addition, an on-site assessor was available at all times to assist where necessary and answer any questions.

### Ethics approval

Ethics approval for this study was granted by the Human Sciences Research Council (HSRC) Research Ethics Committee (REC), protocol approval number REC4/21/08/13. Furthermore, additional approval was also obtained from the Department of Health and Social Welfare, Mpumalanga Provincial Government, and the University of Miami Miller School of Medicine Institutional Review Board (IRB ID: 20130238). Written informed consent was obtained from all participants.

### Randomization

Twelve CHCs were matched in a 1:1 ratio according to patient census and average antenatal clinic patient load, and one clinic in each pair of clinics was randomly assigned to intervention or control using a computer program written by the data manager. The matched clinics were then assigned to the opposite condition. The randomization process was carried out by four people. The first conducted the computer-generated randomization assignments stratified by clinic size (this involved selecting a seed for the random number generator, ran the program, and completed the table of condition assignments). The second implemented the assignments generated by the program, providing a table of all clinic site assignments to study personnel. The third activated each intervention site individually, and the fourth activated each control site individually.

### Blinding

Only the Human Sciences Research Council (HSRC) study staff activating and overseeing the sites were aware of site assignment. All assessments were conducted using an audio computer-assisted self-interview (ACASI) program. As such, participants enter their data themselves and are blind to their assignment. Following randomization, clinic sites were activated individually, and clinic staff were blinded to the condition. Training for clinic study staff was conducted by condition, and clinic study staff conducting the study were also blind to clinic randomization status. Finally, data analysis to evaluate study outcomes was blinded to the clinic’s status as an intervention or control intervention arm.

### Intervention

#### Conditions

Prenatally, participants attended three group intervention (or time-equivalent control) sessions, along with one individual or couples session; postnatally, participants attended two individual or couples sessions. In both conditions, mothers who had not disclosed their HIV status were invited to (1) disclose their status to their partner during a one-on-one couple session, or (2) not have a male partner attend the one-on-one session. Both the control and intervention groups also received the standard of care National Department of Health antenatal PMTCT education sessions.

Intervention. Intervention participants received the PMTCT standard of care plus the ‘Protect Your Family’ intervention led by study-trained clinic staff. The intervention consisted of three prenatal weekly two-hour (between five and seven participants) gender-concordant group sessions and one prenatal individual or couples counselling session, along with two postpartum (6 weeks and 3 months) individual or couples counselling sessions. The ‘Protect Your Family’ intervention is a manualized, closed, structured behavioural risk-reduction programme. The intervention targeted prevention of vertical transmission, adherence to PMTCT and medication use, HIV testing of family members, prevention of HIV transmission and stigma, serostatus disclosure, partner communication, intimate partner violence (IPV), safe infant feeding, safer conception, family planning and dual method sexual barrier use. The intervention sessions for men were designed to be appropriate for partners of both HIV-positive and HIV-negative women, addressing men’s issues, PMTCT, positive family relationships, communication, and child health issues. More details on the intervention are provided in the published research protocol [16].

**Control condition**. Control condition participants received the PMTCT standard of care plus three time-equivalent, group-administered video presentations prenatally. These video sessions covered various topics related to child health, specifically: 1) diarrhea management, dehydration and exclusive breastfeeding, 2) infant nutrition, and 3) immunization and sexual abuse. In addition to the group-administered presentations, control participants attended three further health promotion video presentations administered in an individual or couples setting: one prenatally on 4) fevers, plus two postnatally at 6 weeks and 3 months postpartum on 5) burns and 6) alcohol use.

### Measures

#### Outcomes

Infant HIV status and survival. Infant HIV status at 12 months was assessed via DNA polymerase chain reaction (PCR). Five drops of blood were drawn from the infant by heel stick using a sterile lancet and a Guthrie card with five wells and stored as dried blood spots (DBS). The DBS was collected by trained CHC nursing staff and couriered to an independent laboratory at the University of Cape Town, Division of Pharmacology for confirmation of HIV serostatus. Infant survival status, defined as miscarriage or death, by 12 months postpartum was obtained from clinic records. To analyse the number of HIV-free surviving infants, the number of HIV-infected infants were not included in analyses. To analyse the number of infants who were either HIV-infected or died at 12-months, a variable was created to combine HIV infection or mortality.

#### Predictors

Sociodemographic information. Sociodemographic characteristics included age, level of education, employment status, relationship status, income, and number of children.

Health information. Health-related characteristics included whether participants had been diagnosed with HIV during the current pregnancy, time since HIV diagnosis, time since initiation of ART, whether the current pregnancy was planned, and children’s HIV status.

Partner Information. Partner-related characteristics included partner’s HIV status and disclosure of HIV status to partner.

Depressive symptoms. Depression was assessed using the Edinburgh Postnatal Depression Scale 10 (EPDS-10)[19], a 10-item instrument in which participants rate how often they have experienced symptoms associated with depression in the past 7 days. Scores range from 0 through 30 and the validated cut-off score for South African populations is 12 [20]. A total, continuous score was used for analyses.

HIV stigma. HIV stigma was assessed using the AIDS-Related Stigma Scale (ARSS) [21], a 9-item instrument with statements representative of HIV stigma (e.g., “People who have AIDS should be ashamed”). For each item, participants indicated whether they agreed (score 1) or disagreed (score 0) with the statement given.

Family planning knowledge. Family planning knowledge was assessed using 8 multiple choice questions adapted from the Safer Conception Knowledge, Attitudes & Practices (SCKAP) and Family Planning Survey [22]. Each item’s response was scored as correct (score 1) or incorrect (score 0), with a possible total score of 8 points.

Intimate partner violence. Intimate partner violence (IPV) was assessed using an adapted version of the Conflict Tactics Scales 18 (CTS-18) [23], which included a 9-item partner physical violence subscale and a 9-item partner psychological victimization subscale.

#### Sample size determination, data management and analyses

An *a priori* power analysis was conducted to ensure that an adequate sample size was used to assess intervention, phase, and phase x intervention differences, taking into account expected levels of attrition and mortality, as previously described[16]. Univariate analyses (means and standard deviations) were used to describe women by demographic and psychosocial characteristics. Baseline demographic and psychosocial characteristics were compared by women who had at least one assessment missing compared to women who were retained for all four follow-up time points. These attrition analyses were completed using chi-square tests for categorical variables and *t*-tests for continuous variables meeting distributional assumptions. As needed, Fischer’s Exact Tests or Mann-Whitney U tests were used as alternatives. Variables found to be significantly associated with attrition at *p* < 0.01 were included in all subsequent analyses.

**Fig 1:**
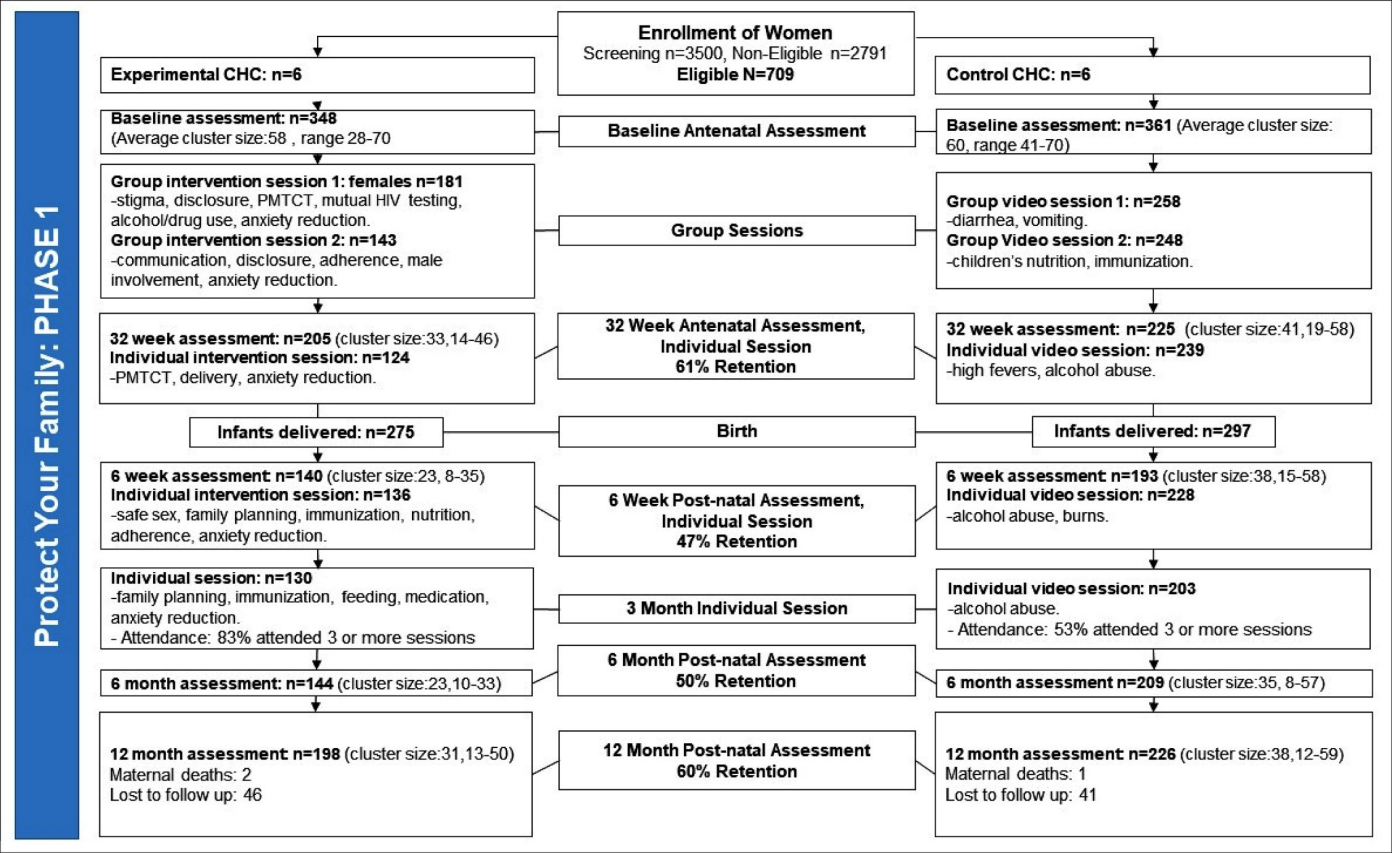
CONSORT diagram outlining enrolment procedures for Phase 1.

**Fig 2:**
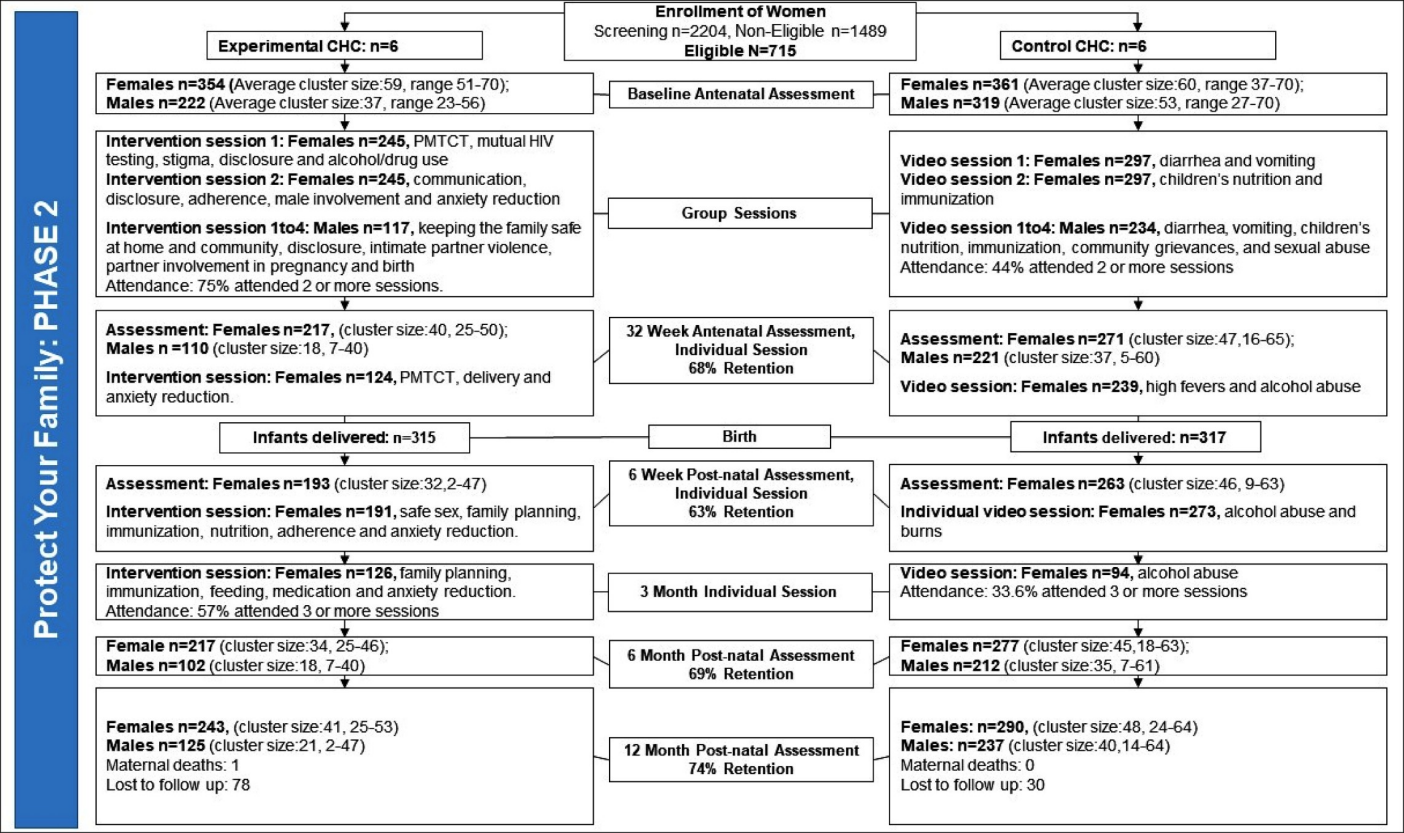
CONSORT diagram outlining enrolment procedures for Phase 2.

To examine the effect of baseline demographic and psychosocial characteristics on rates of HIV infection and survival, unadjusted (bivariate) and adjusted (multivariable) logistic regression models were used. To adjust for the intraclass-correlation for the dependency within community health centers, random effects for the intercept and random slope were estimated. To estimate effect sizes, odds ratios were used [24]. To handle missing data (as reported in Figs 1 and 2), multiple imputation was utilized, specifying ten imputed datasets [25]. Results with or without imputed data were nearly identical, the final model utilized the model with imputed data. All statistical analyses were performed on Mplus (version 8.1) [26].

## Results

### Recruitment and randomization

Originally about 1424 women were identified to be eligible for recruitment into the study. Only about 18 eligible women declined to participate and 7 were lost as a result of technical issues, resulting in a total of 1399 participants at baseline. The Consolidated Standards of Reporting Trials (CONSORT) diagrams are presented in Figs 1 and 2.

### Baseline demographic and psychosocial characteristics of women

Women were a mean age of 28 years (SD = 5.82). Nearly half (48%) of women had completed 10 to 11 years of education, and as part of study eligibility, all women had a partner; 54% were unmarried living separately from their partner, 26% were cohabiting, and 20% were married. Two-thirds (64%) of participants had a monthly income of at least 1000 ZAR (~USD$70). Just over half (55%) of the women had been diagnosed with HIV during the current pregnancy, 50% reported that their pregnancy was unplanned, 79% had at least one child, 27% reported that their partner was HIV-infected, and out of women who reported having children, 6% indicated that they had an HIV-infected child. On average, it had been 24 months (SD = 35.9) since women had been diagnosed with HIV, and 15 months (SD = 25.4) since they had initiated ART. Male involvement and family planning knowledge were moderate, and HIV-related stigma was low. The average EPDS-10 score (i.e., reported symptoms of depression) was 11.7 (SD = 6.01), which translated to 45% of women being above the clinically significant cutoff for depression. Approximately 15% of women reported having more than 2 alcoholic drinks in the past month, and 61% reported having disclosed their HIV status to their partner. Overall, by12 months postpartum, 5% of infants tested positive for HIV and 9% had died. At 12-months postpartum, 91% of infants were alive and HIV-free; 13% were dead or HIV-infected at 12-months. Further detail on the baseline demographic and psychosocial characteristics of women is presented in Table 1.

**Table 1:** Bivariate associations with attrition (N = 1399).

	Total (N = 1399) N (%) Mean (SD)	Lost (n = 877) N (%) Mean (SD)	Retained (n = 522) N (%) Mean (SD)	*t/X*^*2*^, *p*
**Sociodemographics**				
Mean age in years (SD)	28.45 (5.82)	28.19 (5.71)	28.90 (5.98)	**2.21, 0.027**
Education				
0-Grade 9	287 (20.5%)	175 (20.0%)	112 (21.5%)	
Grade 10–11	673 (48.1%)	432 (49.3%)	241 (46.2%)	
Grade 12 or more	439 (31.4%)	270 (30.8%)	169 (32.4%)	1.27, 0.529
Relationship status				
Unmarried, living separate	754 (53.9%)	484 (55.2%)	270 (51.7%)	
Unmarried, cohabiting	368 (26.3%)	219 (25.0%)	149 (28.5%)	
Married	277 (19.8%)	174 (19.8%)	103 (19.7%)	2.34, 0.314
Work				
Unemployed	1072 (76.6%)	680 (77.5%)	392 (75.1%)	
Employed	327 (23.4%)	197 (22.5%)	130 (24.9%)	1.09, 0.297
Monthly income				
< 1000 Rand (USD$70)	498 (35.6%)	348 (39.7%)	150 (28.7%)	
≥ 1000	901 (64.4%)	529 (60.3%)	372 (71.3%)	**17.10, < 0.001**
**Health variables**				
Diagnosed with HIV during this pregnancy				
No	620 (45.3%)	373 (43.4%)	247 (48.4%)	
Yes	749 (54.7%)	486 (56.6%)	263 (51.6%)	3.24, 0.072
Pregnancy unplanned				
No	682 (49.8%)	687 (50.2%)	256 (50.2%)	
Yes	687 (50.2%)	433 (50.4%)	254 (49.8%)	0.05, 0.829
Number of children				
None	298 (20.8%)	198 (22.6%)	100 (19.2%)	
One or more	1101 (78.7%)	679 (77.4%)	422 (80.8%)	2.28, 0.131
Partner HIV positive				
No/unknown	1020 (73.0%)	664 (75.7%)	356 (68.5%)	
Yes	377 (27.0%)	213 (24.3%)	164 (31.5%)	**8.71, 0.003**
Has HIV positive child				
No	1032 (93.8%)	647 (95.3%)	385 (91.4%)	
Yes	68 (6.2%)	32 (4.7%)	36 (8.6%)	1.35, 0.245
Time since ART initiation (months)	15.25 (25.37)	13.30 (23.77)	18.52 (27.57)	**4.10, < 0.001**
Time since HIV diagnosis (months)	24.15 (35.94)	22.14 (34.82)	27.55 (37.54)	**3.25, 0.001**
**Psychosocial variables**				
Male involvement	7.30 (3.19)	7.30 (3.22)	7.28 (3.14)	0.33, 0.745
Stigma	1.27 (1.27)	1.31 (1.34)	1.22 (1.14)	0.55, 0.552
Family planning knowledge	4.48 (1.31)	4.45 (1.34)	4.53 (1.25)	**2.15, 0.032**
Depression	11.73 (6.01)	12.18 (5.93)	10.98 (6.07)	**3.65, < 0.001**
Psychological Intimate Partner Violence	3.46 (6.02)	3.27 (5.99)	3.78 (6.07)	1.75, 0.080
Physical Intimate Partner Violence	1.26 (4.16)	1.23 (4.28)	1.31 (3.93)	0.43, 0.671
Alcohol (> 2 drinks in a day in the past month)				
No	1171 (85.5%)	740 (86.0%)	431 (84.5%)	
Yes	199 (14.5%)	120 (14.0%)	79 (15.5%)	0.61, 0.435
Disclosure of HIV status to Partner				
No	539 (39.3%)	352 (40.9%)	187 (36.7%)	
Yes	831 (60.7%)	508 (59.1%)	323 (63.3%)	2.44, 0.118
Infant HIV status at 12 months				
Negative	1105 (95.3%)	610 (96.1%)	495 (94.5%)	
Positive	54 (4.7%)	25 (3.9%)	29 (5.5%)	1.65, 0.199
Infant alive at 12 months				
No	118 (9.0%)	116 (14.8%)	2 (0.4%)	
Yes	1190 (91.0%)	669 (85.2%)	531 (99.6%)	**79.24, < 0.001**
Infant alive and HIV-free at 12 months				
No	117 (9.3%)	115 (15.2%)	2 (0.4%)	
Yes	1137 (90.7%)	644 (84.8%)	493 (99.6%)	**77.03, < 0.001**
Infant dead or HIV-positive at 12 months				
No	1173 (87.3%)	680 (82.9%)	493 (94.1%)	
Yes	171 (12.7%)	140 (17.1%)	31 (5.9%)	**35.84, < 0.001**

Note. **Bold denotes significant values.** Retained = completed at least one assessment after baseline. Lost = did not complete any assessments after baseline

### Attrition analyses

Attrition analyses using multivariable logistic regression indicated that women who were retained were more likely to be older, have greater income, have an HIV-infected partner, have a greater time since ART initiation and HIV diagnosis, greater family planning knowledge, and lower depressive symptomatology. Comparisons of women who were lost as opposed to retained are presented in Table 1. HIV-infection status was known for *n* = 1159 infants at 12-months postpartum out of *N* = 1399 participants at baseline, and not known for *n* = 240 infants; these were imputed. Alive status was not known for *n* = 91 infants and known for *n* = 1308; therefore, alive status was imputed for n = 91 infants. Either HIV or alive status was known for *n* = 1344 participants out of *N* = 1399; as such, only *n* = 55 were not known.

### Associations with infant HIV infection

Phase 1 had significantly more HIV-infected infants than Phase 2 at 12-months postpartum (aOR = 4.55 [1.38, 15.07]). In addition, increased depressive symptoms were associated with infant HIV infection at 12-months postpartum (aOR = 1.06 [1.01, 1.10]). No other significant associations with infant HIV infection were found (see Table 2).

**Table 2:** Intervention effects on infant HIV infection and survival status (N = 1399).

	Infant HIV Infection at 12 months		Infant Survival at 12 months		
	OR [95% CI]	AOR [95% CI]	OR [95% CI]	AOR [95% CI]	
*Fixed effects*					
Intervention	0.65 [0.37, 1.14]	1.53 [0.06, 39.45]	1.12 [0.77, 1.64]	1.29 [0.38, 4.33]	
Phase	2.89 [1.54, 5.47]*	4.55 [1.38, 15.07]*	0.88 [0.60, 1.28]	0.86 [0.35, 1.71]	
Intervention x Phase	0.42 [0.12, 1.51]	0.06, 3.90]	0.99 [0.46, 2.13]	0.98 [0.45, 2.17]	
*Covariates*					
Age	1.02 [0.97, 1.07]	1.02 [0.97, 1.08]	0.99 [0.96, 1.03]	0.98 [0.94, 1.02]	
Monthly income	1.67 [0.88, 3.16]	1.27 [0.75, 2.17]	1.42 [0.96, 2.10]	1.50 [0.98, 2.31]	
Partner HIV positive	0.54 [0.23, 1.23]	0.52 [0.18, 1.49]	1.31 [0.74, 2.30]	1.22 [0.83, 1.80]	
Time since ART initiation	0.99 [0.98, 1.01]	0.99 [0.98, 1.05]	1.00, 0.99, 1.01]	1.00 [0.99, 1.01]	
Time since HIV diagnosis	0.99 [0.98, 1.01]	1.00 [0.99, 1.02]	1.00 [0.99, 1.01]	1.01 [1.00, 1.01	
Family planning knowledge	0.97 [0.79, 1.21]	1.03 [0.81, 1.31]	1.05 [0.91, 1.21]	1.05 [0.91, 1.21]	
Depression	1.03 [0.99, 1.08]	1.06 [1.01, 1.10]*	0.97 [0.94, 1.00]	0.97 [0.95, 0.99]**	
*Random effects*	B	95% CI		B	95% CI
Intercept	6.00	2.68, 9.32		-2.50	-4.58, -0.41
Clinic	0.93	-0.92, 2.77		0.000	-0.01, 0.01
*Model fit*					
-2LL (Deviance)		471.3		858.9	
Numbers of parameters		12		12	
AIC / BIC		495.28 / 558.46		882.90 / 946.08	
ICC (without covariates)		0.235		0.001	

Note. Unstandardized logistic coefficients were shown. SE = Standard error. CI = Confidence Interval. AIC = Akaike Information Criteria. BIC = Bayesian Information Criteria. Covariances between random intercept and random slope were fixed to 0. ICC = Intra-class correlations.

*p < .05.

** p < 0.01

*** p < 0.001

### Associations with infant survival

Decreased depressive symptoms were associated with infant survival at 12-months postpartum (aOR = 0.97 [0.95, 0.99]). No other significant associations with infant survival were found. Neither intervention nor phase were associated with infant survival at 12-months (see Table 2).

### Associations with infant HIV-free survival

Decreased depressive symptoms were associated with infant HIV-free survival at 12-months postpartum (aOR = 0.96 [0.94, 0.99]). No other significant associations with infant HIV-free survival were found. Neither intervention nor phase were associated with infant HIV-free survival at 12-months (see Table 3).

**Table 3:** Intervention effects on infant HIV-free survival, and HIV infection and mortality at 12 months (N = 1399).

	Infant HIV-Free Survival at 12 months		Infant HIV infection and mortality at 12 months		
	OR [95% CI]	AOR [95% CI]	OR [95% CI]	AOR [95% CI]	
*Fixed effects*					
Intervention	0.78 [0.57, 1.08]	1.27 [0.38, 4.28]	1.16 [0.79, 1.71]	1.29 [0.38, 4.33]	
Phase	1.66 [1.19, 2.31]**	0.78 [0.43, 1.41]	0.82 [0.56, 1.21]	0.86 [0.35, 1.71]	
Intervention x Phase	0.78 [0.40, 1.52]	1.04 [0.49, 2.20]	0.99 [0.46, 1.15]	0.98 [0.45, 2.17]	
*Covariates*					
Age	1.02 [0.99, 1.04[	0.97 [0.93, 1.01]	0.99 [0.96, 1.02]	0.98 [0.94, 1.02]	
Monthly income	0.94 [0.67, 1.32]	1.43 [0.92, 2.23]	1.36 [0.91, 2.01]	1.50 [0.98, 2.31]	
Partner HIV positive	0.67 [0.42, 1.09]	1.50 [0.81, 2.50]	1.35 [0.76, 2.37]	1.22 [0.83, 1.80]	
Time since ART initiation	0.99 [0.99, 1.00]	0.99 [0.99, 1.01]	1.00 [0.99, 1.01]	1.00 [0.99, 1.01]	
Time since HIV diagnosis	0.99 [0.99, 1.00]	1.01 [0.99, 1.02]	1.00 [0.99, 1.01]	1.01 [1.00, 1.01	
Family planning knowledge	0.96 [0.85, 1.09]	1.50 [0.91, 1.19]	1.05 [0.91, 1.22]	1.05 [0.91, 1.21]	
Depression	1.03 [1.00, 1.06]*	0.96 [0.94, 0.99]**	0.97 [0.94, 1.00]	0.97 [0.95, 0.99]**	
*Random effects*	B	95% CI		B	95% CI
Intercept	6.00	2.68, 9.32		-2.50	-4.58, -0.41
Clinic	0.93	-0.92, 2.77		0.000	-0.01, 0.01
*Model fit*					
-2LL (Deviance)		471.3		858.9	
Numbers of parameters		12		12	
AIC / BIC		495.28 / 558.46		882.90 / 946.08	
ICC (without covariates)		0.235		0.001	

Note. Unstandardized logistic coefficients were shown. SE = Standard error. CI = Confidence Interval. AIC = Akaike Information Criteria. BIC = Bayesian Information Criteria. Covariances between random intercept and random slope were fixed to 0. ICC = Intra-class correlations.

*p < .05.

** p < 0.01

*** p < 0.001

### Associations with infant HIV infection or mortality

Phase 1 had a significantly greater proportion of dead and HIV-infected infants than Phase 2 at 12-months postpartum (aOR = 1.98 [1.33, 2.94]). Depressive symptoms were also associated with infant HIV infection and mortality at 12-months postpartum (aOR = 1.04 [1.02, 1.07]). No other significant associations with infant HIV infection and mortality emerged. Furthermore, the intervention was not associated with infant HIV infection and mortality at 12-months (see Table 3).

## Discussion

This study examines male partner involvement in the antenatal period as well as other factors that may impact first year MTCT and survival among HIV-exposed infants. To our knowledge, this is one of the first experimental studies conducted in South Africa to assess the impact of MPI on MTCT and survival amongst HIV-exposed infants. More infants seroconverted in Phase 1 of the study compared to Phase 2. Since Phase 2 enrolled women together with their male partners, this suggests that male engagement in PMTCT improves infant outcomes. It must be highlighted that in the first phase of the study MPI was measured through self-report by women enrolled in the study using a male involvement index. However in the second phase the male partners had to also be enrolled in the study and thus this phase went beyond measuring self-reported MPI and also emphasised actual male partner participation in antenatal care.

Furthermore, women who were recruited in the second phase of the study together with their partners being participants in the study showed much lower rates of attrition and loss to follow-up in the study. Although there was no association between intervention and HIV infection or survival, male participation in the intervention may have promoted greater MPI overall, including in PMTCT and child nurturing, leading to decreased risk of infant HIV infection and mortality. Male involvement therefore should be emphasized in areas with high rates of HIV transmission during or after pregnancy to enhance infant outcomes among HIV-exposed infants.

The results from this study corroborate findings from other studies which found that MPI greatly improves the health outcomes of infants and decreases chance of mortality among HIV-exposed infants. For example, in an experimental study conducted in Kenya, it was found that infants born to mothers without MPI in antenatal care were almost four times as likely to die or seroconvert by 6 weeks postpartum [11]. Similarly, in a study conducted in Ethiopia, MPI was positively associated with infants’ HIV-negative status at 24 months [14]. Several other studies have also reported on some of the general determinants that lead to increased male partner involvement in antenatal care, which may aid in designing of interventions that are aimed at enhancing MPI as part of comprehensive PMTCT programmes [15,27,28]. Our study is unique as it is one of the first to test the effect of male involvement on PMTCT in a clinical trial. This is likely to have an effect not only during pregnancy but to persist after delivery and improve overall health of vulnerable infants born to HIV-infected mothers.

Even though most studies exploring MPI report generally positive perceptions from both male and female participants, the actual rates of men following through are generally low [29,30]. With all the documented evidence of the benefits of involving males in antenatal care, several studies have reported on some of the challenges and barriers that results in lower MPI rates in the SSA region [9, 30, 31]. Health interventions that are targeted at increasing MPI in antenatal care need to address various types of barriers to MPI–including socioeconomic barriers, health systems related barriers, cultural beliefs, and gender roles [9]–both at the health facility level as well as community level. Recent studies conducted in South Africa in the area of MPI in antenatal and maternal care have also provided a useful framework for understanding the sociocultural context and ethnic influences on men’s involvement in maternal and child issues [15,32].

This study also found that antenatal depression in HIV-infected women was associated with perinatal HIV transmission and infant mortality. Although screening for depression is recommended during pregnancy, prevalence of depression screening is suboptimal, including in South Africa [33]. Our results emphasize the importance of establishing effective programmes for depression in order to improve the health of women, and to decrease perinatal HIV transmission and infant mortality.

The present study is not without limitations. The main limitation of this study is that we are reporting significant associations that may not necessarily imply causality. Other limitations included high attrition of study participants, which limited the number of infants who could be tested at 12-months, and those who had a survival status in the clinic records by 12-months postpartum. However this study went a long way in giving a deeper understanding of the socio-economic context of South African communities, on issues around infants and maternal health.

### Conclusion

Male partner involvement in antenatal care is critical in ensuring infant survival and prevention of HIV infection among children born to HIV-positive mothers. In addition, this study highlights the high risk for new-borns associated with antenatal depression and underscores the need of screening for depressive symptoms during pregnancy in order to improve treatment adherence and decrease infant HIV infections and infant mortality.

## Supporting information

S1 Consort Consolidated standards of reporting trials (CONSORT) checklist. (PDF)

S1 Protocol Study protocol. (DOCX)

S1 Data Infant_hfs plos one. (SAV)
