# Supplementary material for: Impact of male partner involvement on mother-to-child transmission of HIV and HIV-free survival among HIV-exposed infants in rural South Africa: Results from a two phase randomised controlled trial
Source: PLoS One. 2019 Jun 5;14(6):e0217467. doi: 10.1371/journal.pone.0217467 (PMC6550447; doi:10.1371/journal.pone.0217467)
Supplement: S1 Protocol — (DOCX) [file pone.0217467.s002.docx]

**Implementing Comprehensive PMTCT and HIV Prevention for South African Couples in Mpumalanga Province**

Proposal

**Prof Karl Peltzer, Prof S Sifunda, S Ramlagan, G Matseke**

HAST,

Human Sciences Research Council,

Private Bag X41, Pretoria 0001,

Tel: 012-3022637; Fax: 012-3022601

E-mail: KPeltzer@hsrc.ac.za

**Prof Deborah Jones &** **Prof Stephen M. Weiss**

Department of Psychiatry and Behavioural Sciences, University of Miami Miller School of Medicine, Miami, Florida, USA

**Significance**

Prevention of Mother to Child Transmission (PMTCT) strategies have dramatically reduced infant morbidity and mortality associated with HIV, as well as significantly improved maternal health (Theuring et al., 2009). Computer modeling estimates indicate that 90-95% participation in PMTCT plus effective pharmacologic regimens (Torpey et al., 2012) would reduce infant HIV incidence to the WHO (2008) goal of < 5% (Ciaranello et al., 2012). Guidelines for antiretroviral (ARV) prophylaxis and combination ARV therapies (cART) to reduce MTCT have been implemented in most HIV-affected countries (UNAIDS, 2012). Despite PMTCT availability, however, not all pregnant women are tested (Van Lettow et al., 2011; Stringer et al., 2008; Larrson et al., 2012; Peltzer et al., 2010b; 2009), not all women receive treatment (Goga et al., 2012), not all mothers provided with medication take it themselves or provide it to their newborns (WHO, 2012; Laher et al., 2012) and not all newborns are tested (SA DOH, 2010; WHO 2012). A meta-analysis of PMTCT studies in 15 sub-Saharan countries also identified differences in PMTCT uptake by pregnant women by method, e.g., HIV opt-out testing uptake in antenatal care exceeded opt-in (94% vs. 58%); for those eligible, ARV prophylaxis coverage exceeded cART treatment (70% vs. 62%); among infants exposed to HIV, early HIV testing exceeded repeated testing at age 12 and 18 months (64% vs. 55%; Wettstein et al., 2012). In addition, pooled analysis of PMTCT adherence studies indicated only an estimated 74% of pregnant women had adequate (>80%) ART adherence; antenatal exceeded postpartum adherence (76% vs. 53%; Nachega et al., 2012).

In South Africa, although significant progress has been recorded in the implementation of PMTCT programs, these improvements have, for the most part, occurred in urbanized areas, with rural areas remaining at unacceptably high levels of MTCT (see also Wettstein et al., 2012). Lack of paternal support, stigma, lack of testing or disclosure, gender dynamics, Intimate Partner Violence (IPV), lack of PMTCT information, clinic access and low retention in care have been identified as major challenges to PMTCT effectiveness in rural areas (e.g., Tabana et al., 2012; Mepham et al., 2011; Bancheno et al., 2010; Bajunirwe et al., 2005; Jones et al., 2005; Rollins et al., 2007; Rutenberg et al., 2003). South Africa’s Mpumulanga Province, the proposed site for this application, is predominantly rural and has one of the highest antenatal clinic (ANC) HIV prevalence rates (37%) in the country. Mpumalanga’s Gert Sibande District reported the highest ANC HIV rate nationwide, 46%, an increase of 7.9% since 2009 (NDOH, 2012). Mpumalanga has one of the lowest rates of PMTCT participation; fewer pregnant women participate in PMTCT services and many (23%) report mixed feeding (Ukpe et al. 2009). While recent reports on South Africa’s PMTCT Programme to reduce transmission to the WHO 2010 goal of < 5% have been promising (Goga et al., 2012), these 2010 PMTCT effectiveness estimates were primarily drawn from urban clinics and did not include “very sick infants” or children who were not immunized at 6 weeks. In contrast, recent Mpumalanga Province infant HIV PCR data at 6 weeks from all clinics in the final quarter of 2011 indicated wide variations in clinic rates of HIV infected infants (5 - 47%, median 13%), higher rates than in 2010, and higher rates of HIV infected infants in follow up testing up to one year (an additional 6 – 22%; National Health Laboratory, 2011). Retention in care remains low (Horwood et al., 2010), infant care may be delayed (Cooke et al., 2009) and recent reports suggest breastfeeding may be responsible for more than half of new pediatric HIV infections (Van de Perre et al., 2012), highlighting the need for behavioral change interventions (NDOH, 2012) to promote safer infant feeding practices (Goga et al., 2011) in combination with ARV adherence (Hudgens et al., 2012). This application is designed to test the efficacy of a piloted intervention strategy to significantly increase uptake of PMTCT by rural HIV positive pregnant women (Peltzer et al., 2011), evaluating the relative contribution of male partners in achieving the goals of PMTCT (<5%) in high MTCT (> 13%) clinics.

**Ante-, peri- and postnatal care & safer feeding.** Two thirds of newborn deaths could be prevented, detected or treated by existing maternal, newborn and child health policy packages, e.g., family planning, PMTCT, skilled birth attendance or clean childbirth, early identification of illness, safer infant feeding, and PMTCT through ARVs (WHO & Partnership for Maternal, Newborn and Child Health; PMNCH, 2006). Yet, mothers may delay ARV onset (Chibwesha et al., 2011) and mothers and newborns often miss postpartum visits and spend most of the postnatal period (0-6 weeks) at home, increasing the risk of maternal or neonatal mortality (Landes et al., 2012; Walker et al., 2012). Attendance at antenatal care may be limited to only one visit (Myer et al., 2012), and poor retention in postpartum care is common (Dube et al., 2012; Kim et al., 2012; Watson-Jones et al., 2012; Chetty et al., 2012). WHO recommends a minimum of four antenatal and four postnatal visits for all HIV positive pregnant women (PMNCH, 2006), e.g., targeting immunizations, interventions, treatment. Mothers may also need additional guidance on infant feeding (Maman et al., 2012) as a single session appears insufficient to sustain the benefits of safer infant feeding (Bequet et al., 2012; Little et al., 2012; Natchu et al., 2012; Ochola et al., 2012). In low and middle-income countries, only 39% of pregnant women received => 4 antenatal visits during 2000–2008, and in Africa, less than half of the poorest 20% of women have access to any antenatal care (WHO, 2012). Adherence to PMTCT protocol at 6 weeks postnatal is low (e.g., Uganda, 38%), and previous adherence to routine care, perception of benefit, access and the presence of spousal support appear to be important motivators for postnatal adherence (Nassali et al., 2009).

PMTCT program failure and dropout occurs at all stages of the ante-, peri- and postnatal process in South Africa (DOH, 2010; Rispel et al., 2009). Implementation of PMTCT programs in overburdened clinics presents multiple challenges, including individual [e.g., denial, depression, maternal failure to ingest medication or provide it to the infant, failure to obtain antenatal or infant testing (e.g., Goga et al., 2012)], interpersonal [e.g., lack of male involvement, intimate partner violence (IPV; Ghanotakis et al., 2012), alcohol or drug use (Tumwesigye et al., 2012)], social [e.g., stigma, (Mepham et al., 2011), lack of disclosure (Kuonza et al., 2011; Turan et al., 2011; 2012; Hardon et al., 2012)], and systemic factors [e.g., home delivery, delayed access to care (Laher et al., 2012)].

**PMTCT uptake and male “involvement”.** Integration of PMTCT with existing services and increasing male participation in antenatal care have been proposed to enhance PMTCT uptake (e.g., Peltzer et al., 2010a, b, c; Conkling et al., 2010). While men are the traditional sexual and reproductive decision makers, the impact of male involvement in PMTCT remains unclear and untested (Montgomery et al., 2011; Brusamento et al., 2012). Taking full advantage of male support may also require a level of communication and cooperation among couples that enables mutual disclosure of HIV status (Dageid et al., 2012). Given the high potential for conflict and IPV, uptake may be limited by lack of HIV status disclosure (Villar-Loubet et al., 2012a; Peltzer et al., 2010d). However, while previous studies were unclear as to what constitutes **effective** male “involvement”(MI), its measurement and its objectives (Mongomery et al., 2012), effective MI is here operationalized as clinic attendance, spousal support, communication, HIV/PMTCT knowledge and HIV testing and disclosure with the objective of facilitating uptake of PMTCT pre- and postnatal practices.

Studies to evaluate the impact of male involvement have been limited (Sherr & Croom et al., 2012), have used varied methods (Byamugisha et al., 2010), and have had mixed results. Media campaigns have been used to increase MI from 4% to 11% (Botswana); couples HIV Counseling and Testing (HCT) has been used to increase MI (Zambia; Wall et al., 2012). MI has increased ANC attendance and HCT (Mohlala et al., 2011), decreased unprotected sex during pregnancy and increased ANC attendance by 9% (South Africa; Mohlala et al., 2011). Increased MI has been associated with nevirapine uptake (Tanzania; Becker et al., 2009), decreased MTCT (Kenya; Alusio et al., 2011) and increased clinic attendance by 15% (Zambia; Katz et al., 2009 a,b). Couples counseling in pre-and postnatal care has been used to facilitate communication about HIV serostatus (Cote d’Ivoire; Desgre’es-Du-Lou et al., 2009), to address barriers to ARV uptake for mothers and their newborns (Kenya: Farquhar et al., 2004; Aluisio et al., 2010), to encourage adoption of safer conception strategies among sero-discordant couples (Orne-Glieman et al., 2010), and to reduce vertical transmission (Kenya; Alusio et al., 2011). In Rwanda and Zambia, couples counseling enhanced follow up but not Nevirapine uptake (Conkling et al., 2010). Despite these results, no trial has definitively tested the effect of MI on PMTCT uptake (Montgomery et al., 2012).

While attendance by couples is feasible (Becker et al., 2009), ANC attendance by men may be “necessary but not sufficient” to influence PMTCT. A review of studies in Africa involving men in antenatal care concluded that male “support” as well as “involvement” may determine increased PMTCT uptake (Auvinen et al., 2010). Men possess general HIV knowledge but lack specific information regarding PMTCT (South Africa; Villar-Loubet et al., 2012) and feel unable to attend antenatal clinics due to work schedules (Tanzania; Falnes et al., 2011). Men also may regard ANC health facilities as being “generally unfriendly” to them (Botswana; Letshwenyo-Maruatona, 2012). Men are perceived as decision makers in the home, and feel their position is undermined if they are expected to attend a “women’s clinic program,” leading them to decline to attend ANC visits with their partners (Orne-Glieman et al., 2010; Theuring et al., 2009; African Development Bank, 2009), with as few as 2% attending ANC (personal communication, Provincial Health Office, Lusaka, Zambia, 2012).

**Disclosure, Stigma and Intimate Partner Violence (IPV).** Following diagnosis of HIV infection, partners may not disclose their HIV serostatus ([Simbayi](http://www.ncbi.nlm.nih.gov/entrez/query.fcgi?db=pubmed&cmd=Search&itool=pubmed_AbstractPlus&term=%22Simbayi+LC%22%5BAuthor%5D) et al., 2007; Vu et al., 2012). Women may fear rejection, abandonment, violence, stigmatization, loss of respect or scapegoating within intimate relationships if serostatus questions are raised (Abrahams & Jewkes, 2012; Sethosa et al., 2005; Bouillon et al., 2007; Deribe et al., 2008; Peltzer, 2011; Rujumba et al., 2012). Men have similar concerns regarding rejection and blaming (Villar-Loubet et al., 2012) as well as exposure of their own sexual behavior (Deribe et al., 2010). The threat of intimate partner violence (Peltzer et al., 2010d; Hyginus et al., 2012; Russell et al., 2012; Shamu et al., 2011; Villar-Loubet et al., 2012; Moses et al., 2012) associated with disclosure during pregnancy may therefore impede access to care during pregnancy and PMTCT uptake (Visser et al., 2008). The initial disclosure of positive test results is one of the greatest stress­ors experienced following an HIV diagnosis (Jones, 1998). Those who anticipate that others will respond supportively may approach both testing and diagnosis with less anxiety and more constructive planning. Those expecting strong negative reactions from others may respond less adaptively (e.g., by social withdrawal) or may avoid receiving test results (SA DOH, 2010). However, both men and women assert that they do not want to be “pushed” to disclose (Villar-Loubet et al., 2012).

In sub-Saharan Africa, IPV has been associated with HIV infection (Ackerman et al., 2002); significant overlap exists between women who are seropositive and those who are battered (Shamu et al., 2011). The prevalence of IPV among pregnant women in South Africa is one of the highest reported globally; risk factors include HIV infection, history of violence, alcohol, and drug use (Shamu et al., 2011; 2012). The ability to disclose HIV serostatus and negotiate safer conception practices is key to controlling transmission, but women are restricted by traditional gender role constraints (Melendez et al., 2003), e.g., antenatally, women with violent or controlling male partners are at increased risk of HIV infection, and more likely to have risky sexual practices imposed upon them during pregnancy (Dunkle et al., 2004). The occurrence of IPV during pregnancy (Makayoto et al., 2012) and gender based power dynamics within couples (Groves et al., 2012; Langen, 2005) have become a serious impediment to accessing PMTCT and to women’s health overall. Our pilot study (Peltzer et al., 2012) identified high rates of IPV among pregnant couples; following intervention, all forms of IPV by men decreased. This application proposes a gender-based group intervention plus couples counseling to directly address IPV, disclosure and stigma, key elements to facilitating PMTCT uptake.

**Safer Conception Practices (SCP), Contraception and Family Planning.** One of the major components of the PMTCT “cascade” is provision of reproductive health choices to enable either the prevention of unintended pregnancies or appropriate planning for intended future pregnancies for women living with HIV (Expert Committee, 2011, DOH, 2010). Many women in sub-Saharan Africa spend the majority of their adult lives pregnant (Mugo et al., 2011) and the majority of women living with HIV are diagnosed during their reproductive years (15- 45 years). As seropositive women initiate or continue childbearing (Westreich et al., 2012; Schwartz et al., 2012; Jones et al., 2010; Harrington et al., 2012), “safer” conception practices [e.g., the use of ART to reduce infectiousness (Cohen et al., 2011), pre-exposure prophylaxis (PreP) to reduce acquisition (Vernazza et al., 2011; Matthews et al., 2012), timed unprotected intercourse (Matthews et al., 2009; Mmeje et al., 2012)], and use of dual contraception (Peltzer et al., 2010; Seutlwadi et al., 2012) are essential to reduce the elevated risk of transmission during conception, pregnancy (Mugo et al., 2011) and postpartum. Complex reproductive strategies and high rates of unplanned pregnancy (Holt et al., 2012; Goga et al., 2012; Peltzer, & Shikwane, 2011) underscore the need to facilitate the integration of PMTCT, safer conception, dual contraception and family planning during ante- and postnatal care (SA DOH, 2010; Lindegren et al., 2012; Schwartz et al., 2012).

Factors limiting the use of safer conception practices are similar to those preventing PMTCT uptake, e.g., fear of HIV disclosure, stigma, and IPV (Taulo et al., 2009), including fears of judgmental healthcare providers with negative attitudes towards childbearing by HIV seropositive women (Barreriro et al., 2007; Peltzer et al., 2009). When women do discuss fertility plans with providers, the extent to which safer conception methods are included is unclear. In Cape Town, while over 30% of HIV positive women wanted additional children, and 60% in Johannesburg planned to conceive in the next year, in both regions, most had never had a conversation with a health care worker on this issue (Schwartz et al., 2012). The proposed intervention utilizes guidelines on safer conception (Bekker et al., 2011) and contraception (Lopez et al., 2010) delivered through combined group, individual and/or couples-counseling interventions to address family planning and the uptake of safer conception practices in conjunction with pre- and postnatal clinical care.

This application addresses limitations in rural PMTCT uptake and proposes a controlled study to evaluate the effectiveness of an intervention to reduce mother to child transmission using prenatal groups and pre- and postnatal individual and couples counseling as well as to assess the impact of male involvement on PMTCT uptake during pre-and postnatal care. Study objectives are a) to maximize PMTCT protocol adherence and retention in care antenatally to 12 months postnatally, and b) to introduce contraception, family planning and safer conception practices into the pre- and postnatal PMTCT protocol. The program extends the existing public health program linking antenatal HIV Counseling and Testing (HCT), PMTCT and family planning services, focusing on reducing barriers to PMTCT that have resulted in unacceptably high levels of MTCT.

**Impact**

Despite the availability of an effective PMTCT treatment protocol and infant feeding guidelines designed for PMTCT, uptake in rural South Africa remains suboptimal (SA DOH, 2011). We hypothesize that PMTCT uptake will be significantly improved by a comprehensive intervention combined with the active engagement and support of male partners in the PMTCT process. Our pilot study was designed to increase male partner involvement in PMTCT to reduce perceived barriers to adherence to the ante-, peri- and postnatal PMTCT protocols. Results confirmed that men will actively participate in an intervention that supports their female partner’s participation in PMTCT and promotes the reduction of sexual risk behaviors and IPV (see Preliminary Studies: Villar-Loubet et al. 2012). While the small sample size of HIV seropositive women in the pilot study precluded definitive conclusions, it provided important guidance and direction for the design of the proposed study and for the refinement of our intervention strategy to target medication adherence, partner communication, mutual HIV status disclosure, reduction of IPV, family planning, safer conception and safer infant feeding practices.

This application seeks to enhance PMTCT uptake pre- and postpartum and reduce MTCT in Community Health Centers in rural Mpumalanga Province, with the goal of reducing current vertical transmission rates of >13% to < 5% among infants at 6 weeks and 12 months of age. Using a sustainable strategy (Peltzer et al., 2010; 2012; Ndou et al., 2012; Uwimana et al., 2012) of training existing CHC staff e.g., antenatal nurses & HCT counselors, to provide counseling in both group and individual sessions tied to regular antenatal and postnatal clinic visits, the application integrates sexual health, family planning and contraception, the prevention of unintended pregnancies as well as planning for intended future pregnancies, one of the goals of the PMTCT “cascade” (Expert Committee, 2011, SA DOH, 2010; Harrington et al., 2012; Goga et al., 2012). Implementation of the intervention by CHC personnel provides the foundation for rapid translation and scale-up of the program. If successful, the “Vikela Umndeni: Protect Your Family” intervention will provide a generalizable integrated, sustainable model for clinics with high rates of HIV and high incidence of MTCT to optimize PMTCT program delivery and effectiveness, which would have major health policy implications for containing the epidemic in two of the most vulnerable affected populations in rural South Africa: HIV seropositive pregnant women and their infants.

**Innovation**

While there have been studies attempting to illustrate the contribution of male participation to PMTCT uptake, as well as behavioral interventions to promote the PMTCT process, this application proposes to capitalize on the findings of our recent efficacy pilot study to determine the relative effectiveness of both strategies, individually or collectively, in promoting PMTCT uptake in rural South Africa. This full factorial, controlled study design will test the effectiveness of the piloted behavioral intervention to significantly increase PMTCT uptake among rural HIV positive pregnant women, simultaneously determining whether the intervention conducted both with or without the participation of male partners will have an additive or synergistic impact on PMTCT uptake. The intervention will utilize a combination of both group and individual or couples counseling strategies, ante – and postnatally, with a 12 month followup assessment of infant serostatus. During antenatal care, the intervention will use a group format to address key issues related to enhancing PMTCT uptake, including PMTCT information, HIV disclosure, coping with stigma, intimate partner violence, adherence to the overall PMTCT protocol and safer conception practices. Just prior to birth and postpartum, the intervention will shift to a individual or couples-based counseling format, targeting medication adherence, safer infant feeding, family planning and safer conception practices (Desclaux & Alfieri, 2009; Makanani et al., 2009). As a goal of the PMTCT protocol, the intervention will facilitate the uptake of safer conception practices by integrating and enhancing the role of health care providers in the delivery of family planning services for women or couples living with HIV.

**Preliminary Studies/Investigators**

This application is based on the extensive HIV research experience of our team (Drs. Jones, Peltzer, Weiss, Mwinsongo) in the US and international settings on medication adherence, PMTCT, and sexual risk reduction among HIV negative and seropositive men and women and serodiscordant couples. Drs. Jones and Peltzer led the PEPFAR PMTCT pilot study in South Africa; Dr. Peltzer is an expert on PMTCT in South Africa; Drs. Weiss and Jones are co-creators of the behavioral risk reduction intervention proposed in this application.

**Promoting Male Involvement to Improve PMTCT Uptake, PI D Jones, CoPIs K Peltzer, S Weiss (NIH/PEPFAR P30AI073961-S).** This one year pilot project (n = 239 couples) provides the foundation for this application. Although the number of women with HIV (n = 76) was too small to derive meaningful statistical comparisons on most findings, the clinical outcomes were sufficiently compelling to provide guidance and direction to the development of this application. Pregnant women and their partners from antenatal clinics in Mpumalanga Province, South Africa were followed from month 4 of pregnancy to 3 months postpartum. Project aims were to increase male involvement in ANC to promote PMTCT and to reduce unprotected sex during pregnancy. Men participated in both experimental and control conditions. The experimental condition included a gender-concordant group intervention based on sexual risk reduction and PMTCT promotion, and the attention-control condition included time-matched usual antenatal care. Baseline findings identified multiple sex partners, high levels of unprotected sex (50%) and IPV among both men and women, and low levels of knowledge about HIV transmission and PMTCT among men (Peltzer et al., 2011). Post-intervention, men had high rates of ANC attendance in both conditions (Experimental: 86%; Control: 79%). Experimental participants decreased unprotected sex at follow up in comparison with control participants (F = 7.16, p = .008). Adjusting for baseline levels, rates of unprotected sex at post-intervention were 32% and 83%, respectively, in experimental and control conditions; mixed estimates of unprotected sex using regression pretest-posttest, adjusting for baseline values, indicated an intervention effect estimate of -0.511, i.e., a 51% reduction in rates of unprotected sex, intervention versus control, assuming an adjusted intra-class correlation (ICC) of 0.14. Qualitative data from both genders reflected concerns about serostatus disclosure and communication (Villar-Loubet et al., 2012a). There was an increasing trend in positive communication and a decrease in the number of sex partners (Villar-Loubet et al., 2012b). Four infants were HIV+ by PCR at 6 weeks (1 experimental, 3 control). In the experimental group, HIV-related knowledge increased (F(1, 451) = 23.52, *p* < .001) and intimate partner violence by men and negative communication by women decreased (F = 6.94, p = .009; F = 9.30, p = .003). Mixed infant feeding, not an intervention target in this pilot study, was practiced by 40% of women. The current application will promote safer feeding practices, i.e., exclusive breast feeding or replacement feeding, and continue to target attendance, sexual risk reduction and safer conception practices.

**Programme to Improve Implementation of the Prevention of Mother to Child Transmission of HIV in Cacadu District of the Eastern Cape and in Gert Sibande and Nkangala Districts of Mpumalanga Province, South Africa. PI, K Peltzer (Overall PI L Simbayi) (CDC-PEPFAR: USG PS000570).** This project aimed to improve PMTCT adherence by evaluation of PMTCT program activities. Identified gaps in PMTCT delivery: Low clinic uptake of Nevirapine (56%). Missed opportunities: HCT among 67% women during pregnancy. Shortcomings identified included health policy, service delivery & health-seeking behavior (Peltzer et al., 2010); other factors identified included high levels of traditional and complementary health practices (Peltzer et al., 2009), IPV and HIV risk (Rispel et al., 2009), barriers to HIV testing (Peltzer et al., 2010b), problems with infant feeding and testing (Ladzani et al., 2011; Peltzer & Mlambo, 2010a) and adherence to ARV prophylaxis (Peltzer et al., 2010c; Peltzer et al., 2011), lack of family planning services (Peltzer et al., 2009), and problems with HCT provision (Peltzer et al., 2011). An intervention was used to strengthen PMTCT services and awareness. Long clinic waits and short counseling sessions remained (Rispel et al., 2009).

**Improve Capacity of an Indigenous Institute to Enhance Monitoring & Eval. of HIV/AIDS in SA, PI, K Peltzer (CDC-PEPFAR: USG PS000570).** This project sought to stimulate male participation in PMTCT in Mpumalanga using community campaigns with male peer mentors in 3 sub-districts. Community mobilization, i.e, door to door campaigns, CHC health talks, were conducted over 6 months; 3300 men were recruited. Only 6% of men attended ANC with partners, 2% also attended HCT with partners. Couples (n = 170) participated in a 3 session program (Peltzer et al., 2011). Barriers included lack of weekend clinic access for working men, disconnect from the “maternal” program, concern about HCT, partner disclosure and gender specific services.

**Research and intervention for prevention-of-mother-to child transmission in a resource poor setting. PI, K Peltzer (Ford Foundation).** This study identified barriers to care and provided health systems support interventions. Factors influencing PMTCT adherence and outcomes were identified (Peltzer et al., 2006, 2007, 2009, 2010c). Intervention studies found positive impact of PMTCT interventions, e.g, training of traditional birth attendants (Peltzer, 2006), maternal self-medication with nevirapine tablets at onset of labor, and maternal provision of nevirapine syrup to newborns (Peltzer et al., 2008).

**Efficacy of a lay health worker led group antiretroviral medication adherence training among non-adherent HIV-positive patients in KwaZulu-Natal, South Africa. PI K Peltzer, CoPIs, D Jones S Weiss (TIBOTEC REACH Initiative).** The effectiveness of lay health worker-led groups to improve ART adherence in adults was assessed in a randomized controlled trial at a district hospital HIV clinic in Mpumalanga. Low adhering patients on ART (n = 152) attended three group sessions plus standard of care, or standard of care alone. Adherence knowledge pre- to post increased in the intervention condition compared to standard of care. Adherence and CD4 count increased and depression decreased in both conditions (Peltzer et al., 2012).

**Interventions to Enhance HIV Medication Adherence in Zambia. D Jones, PI, I Zulu, S Weiss, N Chitalu, Co PIs (R21AI067115)**. This clinical trial sought to increase adherence among naïve ARV users in Zambia (n=140) utilizing a 3 session psychoeducational group; most participants reported 100% adherence; 15% reported non-adherence over 3 months. Pharmacy records, pill count, patient report and provider evaluation were triangulated to evaluate adherence. The number of sessions attended, drop out rate and health care appointments attended did not differ between conditions, while participants in the intervention condition reduced the number of missed doses and maintained the reductions over time (F = 2.37, p = .04) in comparison with the individual condition (Jones et al., 2012).

**Barrier Acceptability among Culturally Diverse HIV+/- Women and their Partners (The NOW/NOW2 Projects), PI S. Weiss, CoPIs N Chitalu, D Jones (NIMH RO1MH63630).** This study enrolled 1080 HIV + and HIV- high risk women in Miami, USA and Lusaka, Zambia and sought to reduce risk of HIV/STD transmission, infection, and re-infection among HIV+/- women (Jones et al., 2001, 2004, 2007). The four weekly session intervention increased barrier acceptability and acceptability predicted use in both US (F(8,40) 3.79, p = .002) and Zambia (F(8,66) 3.21, p = .004). Participants increased use and acceptability of female condoms and maintained condom use at 6 and 12 months. Intervention participants increased male condom use at 6 months (reduced to non-significant “trend” at 12 months) and reduced sexual risk behavior (Jones et al., 2006).

**Implementing HIV Risk Reduction in Zambia (Partner/Partner 2 Projects), PI D Jones, Co-PIs S Weiss and N Chitalu, (NICHD/NIH R24HD43613; R01HD058481).** This clinical trial (n= 420 Zambian couples) of a four weekly session couples’ sexual behavior intervention found that women in the intervention had higher condom use (F = 5.68, p = .02), more positive condom attitudes, increased plans for safer sex, and less alcohol use (Jones et al., 2005); female and male condom use increased at 6 months and was maintained over 12 months (Jones et al., 2008a). Communication influenced condom use, and use increased among inconsistent condom users in both conditions and was maintained over 12 months. HIV- men increased barrier use (*F* = 16.13, *p* =.001; Jones et al., 2008b). The study was translated to 6 Lusaka CHCs (n = 420 seropositive & seroconcordant couples); at 12 months follow-up couples had higher sexual barrier use (F = 7.17, p = .001; Jones et al., 2009; 2010; Vamos et al., 2012), couples achieved and sustained comparable positive results. The intervention is being disseminated nationwide in Zambia (Weiss et al., 2011).

**Specific Aims.**

Despite the availability in South Africa of effective prevention of mother to child transmission (PMTCT) treatment and infant feeding guidelines to prevent vertical transmission of HIV, not all pregnant women are tested (Van Lettow et al., 2011; Stringer et al., 2008; Kieffer et al., 2010; Peltzer et al., 2010b; 2009), not all women receive treatment (Goga et al., 2012), not all mothers provided with medication take it themselves or provide it to their newborns (WHO, 2012), and not all newborns are tested (SA DOH, 2010; WHO 2012). Implementation of PMTCT programs in already overburdened clinical settings presents structural, social, individual and interpersonal challenges (e.g., Rispel et al., 2009), and these limitations are particularly evident in rural communities in South Africa. Similar to other African nations attempting to integrate PMTCT into pregnancy and infant care, efforts to scale up and reduce transmission to <5% in South Africa have fallen short, particularly in rural settings (Wettstein et al. 2012). Mpumalanga Province, a predominantly rural region and the focus of the current application, has one of the lowest rates of PMTCT uptake in South Africa (Shisana et al., 2010): 69% in 2009, with a 2011 HIV antenatal clinic (ANC) prevalence of 40.6% and infant HIV rates as high as 50% in Mpumulanga’s rural ANCs (NDOH, 2012; National Health Laboratories, 2011).

Although increasing male participation as a strategy to enhance uptake of and commitment to the PMTCT protocol for pregnancy, newborn care and family planning has been identified as a potentially critical component of PMTCT programs (e.g., Peltzer et al., 2010a; Conkling et al., 2010; **Wettstein et al., 2012**), the true impact of male involvement remains unclear and untested (Montgomery et al., 2011; Brusamento et al., 2012). This application proposes to test the effectiveness of an intervention to significantly increase uptake of PMTCT by HIV positive pregnant women, while evaluating the additional contribution of male partner involvement on PMTCT outcomes. Building upon the promising pilot results from our PEPFAR/NIAID Miami DCFAR supplement (P30AI073961), we propose to expand the comprehensive risk reduction, medication adherence and PMTCT intervention to 2160 study participants in predominantly rural districts of Gert Sibande and Nkangala in Mpumalanga Province. The pilot PMTCT study successfully engaged men in the ANC and PMTCT process, increasing PMTCT knowledge and positive communication, reducing IPV and sexual risk behaviors (see Preliminary Studies). The proposed study will recruit HIV positive pregnant women and partners, and enroll 720 HIV positive pregnant women (Phase 1- Women only) and 720 HIV positive pregnant women plus their male partners (Phase 2- Couples) from 12 Community Health Centers (CHCs) with high rates of vertical transmission, i.e., > 13% MTCT rates. Using a cluster-randomized design, CHCs will be randomized to experimental and control conditions to test the impact of the intervention, with or without male participation, to improve PMTCT uptake and postnatal infant medication provision and feeding practices.

**Specific Aim 1**: to increase uptake and adherence to ante-, peri- and post-natal PMTCT protocols by HIV positive pregnant women through the implementation of a comprehensive, evidence-based risk reduction, medication adherence and PMTCT intervention.

**Hypothesis 1.1:** Within each Phase, Experimental condition mothers will be significantly more likely to adhere to PMTCT protocol medications as prescribed compared to Control condition mothers at 32 weeks pregnant, and 6 and 12 months postpartum, and provide them to infants up to 6 weeks.

**Hypothesis 1.2:** Within each Phase, infants born to Experimental condition mothers will be less likely to test HIV+ at 6 weeks and 12 months of age compared to those born to Control condition mothers.

**Specific Aim 2**: to retain women and infants in post-natal care to ensure adherence to PMTCT safe infant feeding protocols (SA DOH, 2010).

**Hypothesis 2:** Within each Phase, experimental condition mothers will be more likely to continue PMTCT safe infant feeding at 6 months and more likely to be retained in care at 6 and 12 months post-partum compared to control condition mothers.

**Specific Aim 3**: to improve sexual and reproductive decision making and safer conception practices.

**Hypothesis 3:** Within each Phase, Experimental condition mothers will decrease unsafe sexual practices and/or increase and maintain the use of safer conception practices (e.g., dual barrier methods) at 32 weeks pregnant and 6 and 12 months post-partum compared to Control condition mothers. This will be accomplished with no increase in intimate partner violence.

**Specific Aim 4**: to assess the impact of male engagement on PMTCT uptake (medication adherence, feeding practices, clinic attendance), safer sex and family planning practices.

**Hypothesis 4:** Within the Experimental condition, mothers in Phase 2 (couples arm) will a) be more likely to take PMTCT medications at 32 weeks pregnant and 6 and 12 months postpartum, and provide medications to their infants up to 6 weeks; b) be more likely to continue safe infant feeding and be retained in care at 6 and 12 months; and c) be more likely to increase safer sex and/or safer conception practices at 32 weeks pregnant, and 6 and 12 months in comparison with mothers in the Experimental condition Phase 1 (women-only arm).

**Approach**

**Design.** This study is a group-randomized controlled trial using a 2 x 2 x 4 comparison Phase (Women-only, Couples) x Condition (Experimental, Control) x Time (Baseline, 32 weeks pregnant, 6 and 12 months postpartum). Community health centers (n = 12 CHCs) in communities with high rates of vertical transmission (>13%) within the Gert Sibande and Nkangala Districts in Mpumalanga Province, South Africa, will be stratified by size and randomly assigned to condition in a 6:6 ratio. The 6 experimental condition CHCs will provide PMTCT plus the “Vikela Umndeni” (“Protect Your Family”) intervention, the 6 control condition CHCs will provide the PMTCT standard of care plus a time-equivalent attention-control condition on childhood disease prevention. Study participants (n = 2160) will be 720 women in Phase 1 and 720 couples (n = 1440) in Phase 2. [N.B. Treatment protocols are in accordance with South African Clinical Guidelines for PMTCT.]

**Experimental condition:**

**Phase 1** (women-only), the 6 experimental clinics will offer the standard of care (PMTCT), plus an intervention to HIV+ pregnant women (n = 360)

**Phase 2** (couples), the 6 experimental clinics will offer the standard of care (PMTCT), plus an intervention to HIV+ pregnant women and their male partners (n = 360 couples).

**Control condition:**

**Phase 1** (women only), the 6 control clinics will provide the standard of care (PMTCT), plus childhood health videos to HIV+ pregnant women (n = 360)

**Phase 2** (couples), the 6 control clinics will offer the standard of care (PMTCT), plus childhood health videos to HIV+ pregnant women and their male partners (n = 360 couples).

**Study Duration.** This study is expected to start in 1^st^ January 2014 and will end in June 30^th^ 2018

**Sites** Study sites will be selected from Gert Sibande District (pop. 890,699, 71 PMTCT sites) and Nkangala District (pop. 1,020,592, 80 PMTCT sites) in Mpumalanga Province, the province with the highest rates of antenatal HIV prevalence in South Africa, i.e., 35.6% in 2009, 35.1% in 2010 (SA DOH, 2010, 2011). Gert Sibande and Nkangala districts have had the highest antenatal HIV prevalence (35.6%, 30.1%) and lowest rates of HIV testing for infants (20.7%, 43.1%) in Mpumalanga [Health Systems Trust, (HST) 2009]. Only 70% of those mothers identified as seropositive took Nevirapine for PMTCT (SA DOH, 2010). Low rates of HIV testing during pregnancy (74.6%, 86.3%) were also found in 2009. While recent reports noted decreasing rates of mother-to-child transmission in South Africa, these decreases are predominantly occurring in urban areas rather than rural locales, such as Mpumalanga Province.

**Study Flowchart**

Clinic sites will be matched on size and then randomized. All sites will meet South African criteria for PMTCT sites: on-site daily HCT; ARV distribution & CD4 testing; antenatal counseling on infant feeding; postnatal counseling and infant feeding support, infant formula; HIV infant testing; > two trained PMTCT staff and 2 counselors; support group for HIV-positive mothers and pregnant women. There is wide variation in MTCT rates (5% - 47%) among the 27 clinics described in the Resources and Environment Clinic Sites section. The MTCT rate mean = 17%, median = 13% for all 27 clinics. Sites selected will be from clinics in the upper 50%, i.e., > 13% MTCT rates, with sufficient numbers of HIV seropositive women receiving antenatal care to achieve recruitment goals.
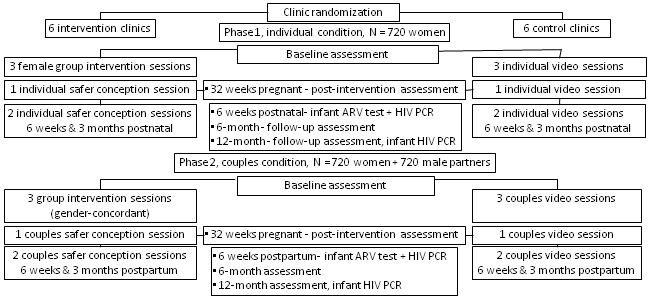


**Participants.** This study will recruit 720 HIV positive pregnant women and 720 HIV positive pregnant women and partners (couples) over 30 months (see Study Flowchart). Each CHC will recruit 60 women, followed by 60 couples over 30 months.

**Eligibility.** Participants recruited will be HIV seropositive pregnant women with partners, 24-32 weeks pregnant (typical time of entry into ANC care), aged >18. In Phase 1 (women only), only women will be enrolled in the study. In Phase 2 (couples phase), both women and their partners will be enrolled. For the purposes of this study, primary male partners are defined as 1) husband, 2) current baby’s father, or 3) current sexual partner.

**Recruitment.** Per South African PMTCT Protocol, all women receive pre- and post-HIV counseling and testing (HCT) and, if HIV+, referral for CD4 assessment and ART; study-eligible women candidates will be referred to the study assessor by PMTCT/HCT staff post-HIV testing and referral for treatment. Seropositive women with partners who have completed HCT will be approached to participate in the study; male partners in the couples phase will be invited to attend by their female partners following initial contact with the recruiter. Those agreeing to participate will be offered an appointment, and enrolled following provision of Informed Consent, maintaining confidentiality of the serostatus of both couple members. Per the standard of care, male partners will be encouraged, but not required, to undergo HCT.

**Site Preparation.** Ethical approval will be obtained prior to study onset. Dr. Peltzer, Dr S Sifunda and the Project Director, will review the objectives of the study and discuss strategies for implementation within the existing CHC framework. CHC site staff (e.g., managers, nurses, HCT counselors) will be briefed on the assessments and intervention at their clinic.

**Site staff Recruitment, Assessment and Behavioral Intervention Training**. Study personnel (counselors and assessors) will undergo formal training on recruitment, assessment, research ethics and intervention procedures by the UM investigators (Jones, Weiss), HSRC intervention trainer (Matseke) and assessment trainer (Ramlagan). Experimental condition leaders will receive intensive training on the intervention and use of audio computer assisted self interview (ACASI) technology, including a five day workshop and guided training and practice under the supervision of experienced interventionists. Training will include in-depth review of the meaning of each item in the assessment instruments presented by ACASI, intensive review of the intervention “Vikela Umndeni” manual, the PMTCT protocol, and use of cognitive behavioral intervention strategies used in the intervention as well as how to manage sensitive issues (e.g., disclosure, IPV, gender dynamics, sexual risk reduction, safer conception practices). Training in the behavioral intervention strategies related to enhancing couple communication, medication adherence per the PMTCT protocol, sexual negotiation, couple disclosure of HIV status and reproductive health will be guided by the manuals created during the pilot study. Training includes practice for facilitation and support for HIV serostatus disclosure during counseling sessions. Following the workshop, each group leader will conduct two series of group sessions and couples counseling sessions under the supervision of the HSRC trainer (Matseke). “Vikela Umndeni” manuals will be adapted for individual women during project onset (see Formative Development). Additionally project team staff will be trained in research ethics through a online NIH course.

Control condition leaders will receive an identical 1 day training session on use of ACASI technology. In addition, leaders will receive a 4 hour orientation to enable them to conduct time-equivalent group sessions comprised of childhood disease prevention videotapes (e.g., measles, diarrhea management, immunizations).

**Quality Assurance.** Quality control will be overseen by project staff, and intervention fidelity will be maintained by audio recording of intervention sessions, interventionist checklists and weekly review by Dr S Sifunda. Evaluation will include review of a random sampling of 10% of the intervention audio recordings and weekly feedback to group leaders. Research staff with bilingual target language skills will transcribe the recorded sessions using headphones in private rooms at the HSRC offices. Dr. Jones will also conduct QC of a 10% sample of transcribed sessions and provide monthly feedback to Dr S Sifunda. Ongoing contact with CHC staff will allow SA team members to respond to questions on study protocol and to discuss and resolve any difficulties that may arise. Monthly conference calls between US and South Africa will address clinic issues including fidelity to protocol and resolution of problems (see Leadership Plan). Semi-annual visits by Miami study investigators will include review of protocol implementation with Dr. Peltzer, Dr S Sifunda, study personnel and CHC staff.

**Formative Development:** **Adaptation of Vikela Umndeni.** The PMTCT and safer conception components of the intervention were developed during our pilot study and earlier studies in Zambia (e.g., Jones et al., 2004), incorporating cultural factors that influence knowledge, attitudes and perceptions about conception and PMTCT. Core issues identified (Villar-Loubet et al., 2012a, 2012b; Peltzer et al.,2012) were integrated into the intervention sessions and reviewed by Dr. Peltzer and his team for cultural congruence. During the initial 3 months of the award, “Vikela Umndeni” will be collaboratively adapted by US and SA teams for the women-only arm (Phase 1) and the content of the couples sessions will be reviewed and refined, guided by experiences from the pilot study (e.g., Jones et al., 2013**;** Villar-Loubet et al., 2012a, 2012b;) as well as input from Drs. Peltzer and Mwisongo on SA cultural issues and from Drs. Jones and Weiss on strategies from earlier “women-only” and couples studies in Zambia (e.g., Jones et al., 2008c, 2010).

**Assessments.** (see Appendix B, proposed Audio Computer Assisted Self Interview (ACASI).

The majority of study materials, e.g., consent, assessment and intervention, were translated into local languages (Ndebele, Zulu & Swati) during the pilot study. Participant assessments include both psychosocial assessments, biological and clinic data at study entry, pre-delivery (32 weeks pregnant), 6 weeks, and 6 & 12 months post-natal follow up. All assessments target PMTCT objectives and will be presented using ACASI technology to enhance disclosure and account for literacy issues. Participants will receive SA Rand 50 (~US$6) per assessment for their time and transportation, and be provided snacks during sessions. All study visits (enrollment, assessments & sessions) will also be offered Saturday afternoons to enhance male participation.

**Biological Assessment & Clinic data.**

**HIV Status, clinic attendance & retention data:** Clinic data include 1) infant PCR HIV test at 6 weeks (clinic data) and 12 months (study administered HIV test); 2) HIV maternal serostatus baseline and 32 weeks pregnant (clinic data); 3) paternal serostatus baseline and at 12 months post-delivery (optional testing, clinic data), and 4) PMTCT clinic visits (maternal, paternal, infant) and study session attendance. PMTCT retention will be dichotomized based on WHO guidelines (2006): Attendance at > 4 prenatal and > 4 postpartum clinic visits and compared to < 4 prenatal and < 4 postpartum.

**ARV uptake assessment**. Maternal adherence will be assessed by blood sampling (dried blood spot, DBS) pre-delivery (32 weeks pregnant); infant adherence will be assessed at 6 weeks by DBS. DBS will assess presence of ARVs for those on ART lifelong medications (Tenofovir, Lamivudine, Emtracitabine, Stavudine, Lopinavir, Retonivir, Efavirenz) and those on short term PMTCT ARVs (Nevirapine, Zidovudine). Samples will be analyzed to obtain a quantitative assessment of the presence of ARVs.

**Self-reported adherence – Mother & Infant** is assessed using a self-report or maternal reported rating of adherence over one month, combined with a **Visual Analogue Scale** (VAS), used to assess adherence over the previous 7 days. Self-reported adherence will be dichotomized as 100% adherent and <100% adherent.

**Demographics** scale is used to assess age, ethnic group, education, income, marital/current partner serostatus, children, children’s serostatus, living situation, family planning and health care use.

| Domain | Instruments | TIME (min) | Base-line | 32 weeks  pregnant | 6 weeks Post-Natal | 6 mo Post-Natal | 12 mo Post-Natal |
| --- | --- | --- | --- | --- | --- | --- | --- |
| HIV status | HCT: Mother (entry eligibility)  HCT: Father (optional)  PCR: Infant |  | X  X | X | X |  | X |
| Retention in Care | Clinic record abstraction | 0 |  |  |  | X | X |
| “Lifelong” ART & ARV Prophylaxis  Adherence: Mother  Adherence: Infant | Dried Blood Spot: Mother  Dried Blood Spot: Infant Adherence rating &  Visual Analogue Scale 1 week: Mother & Infant | 5  5  5 | X | X  X | X  X | X | X |
| Demographics | Demographic Interview | 5 | X |  |  |  |  |
| Stigma | HIV/AIDS Stigma Instrument | 5 | X |  |  |  | X |
| HIV Disclosure | Disclosure Scale | 5 | X | X |  | X | X |
| IPV & Communication | Conflict Tactics Scale | 5 | X | X |  | X | X |
| HIV & PMTCT Knowledge | HIV & PMTCT Knowledge Questionnaire | 5 | X | X |  | X | X |
| Contraception & Safer contraception practices | Sexual Barrier Use Diary  Safer Conception KAP  Reproductive Decision-Making | 5  5  5 | X  X  X | X  X  X |  | X  X  X | X  X  X |
| Infant Feeding | Infant Feeding Diary | 5 |  |  | X | X | X |
| Male engagement | Male Involvement Index | 5 | X | X |  | X | X |

**Stigma** is assessed using an adaptation of the **HIV/AIDS Stigma Instrument – PLHA** (Holzemer et al., 2007) and measures perceived and enacted stigma in the home, community, workplace and health care settings. Stigma is conceptualized as internal (e.g., self-exclusion from services and opportunities, social withdrawal, and fear of disclosure) and external (e.g., avoidance, rejection, and abuse).

**HIV Disclosure** is assessed using an adaptation the **Disclosure Scale** (Visser et al., 2008) assessing disclosure among sexual partners and family members during pregnancy, and factors associated with disclosure, knowledge of partners’ HIV status, and previous HIV counseling or education.

**IPV & Communication** are assessed using an adaptation of **Conflict Tactics Scale** (Strauss et al., 1979), an 18-item scale which assesses conflict resolution style over the current month and previous 12 months, in three domains of positive and negative interactions and violence.

**HIV & PMTCT Knowledge is** assessed using an adaptation of the **AIDS-Related Knowledge Test** (Fisher et al., 1996). This adaptation from the 10-item scale assesses HIV risk and prevention-related knowledge. Items reflect information about HIV transmission, condom use, AIDS-related and PMTCT-specific knowledge and are responded to as Yes, No, or Don’t Know. The AIDS knowledge test is scored for the number of correct responses. The AIDS knowledge test has acceptable internal consistency (alpha = .73).

**Sexual Barrier Use** is assessed using the **Sexual Barrier Diary,** which asks participants to recount their sexual activities, indicating for each day of the past week whether or not they had intercourse, and if so, the type of sexual barrier method used, if any.

**Safer Conception Knowledge, Attitudes & Practices (KAP) & Family Planning** is assessed using an adapted **SCKAP** **Survey** on knowledge, attitudes and use of safer conception practices, including individual, interpersonal, sociocultural, structural factors (Idonije 2011). **Reproductive Decision Making** is adapted from Dommering et al. (2011) for women living with HIV, and assesses current and previous fertility intentions (FI), and factors associated with FI (e.g., perceived risk of transmission, perceived burden).

**Infant Feeding** is assessed using an adaptation of the WHO (2001) feeding scale measuring breastfeeding and replacement feeding practices used in the context of PMTCT in the last 7 days.

**Male Engagement** is assessed from clinic attendance records and an adapted form of the Male Involvement Index (Byamugisha et al., 2010; Peltzer et al., 2011), a 6-item scale assessing male ante-natal involvement, as well as group and couples counseling attendance.

**Intervention** **Condition**

**Model.** The intervention applies the Information Motivation Behavioral Skills (IMB) model to the objectives of the PMTCT protocol (PMTCT related-information, motivation to engage in PMTCT, and behavioral skills to prevent transmission), to promote PMTCT uptake (Fisher et al., 2006). The intervention provides Information on PMTCT (realistic expectations for medication, side effects, treatment duration, the relationship between PMTCT, ARVs, viral load, CD4, infant feeding and HIV transmission) that enhances treatment Motivation (positive attitudes on medication and engagement in care and treatment) and Behavior (increased health care attendance behaviors, coping with stigma and disclosure) to increase adherence to treatment and retention in care. This process promotes positive health outcomes and provides Information (e.g. prevention of MTCT, viral load suppression, subjective perceptions of improved health, reduced distress) that increase Motivation to maintain adherence Behavior to the PMTCT protocol over time (Fisher et al., 2008). The IMB model has been applied in a variety of HIV+ and chronic condition patient populations and settings (e.g., Amico, 2011; Starace et al., 2006; Amico, 2006; Fisher et al., 2006), including those with limited health literacy (Fisher et al., 2008).

**Vikela Umndeni”(Protect Your Family)** **Intervention plus PMTCT Standard of Care (SOC).**

“Vikela Umndeni” intervention is a manualized, closed, structured behavioral risk reduction program targeting HIV, stigma, disclosure, communication, IPV, PMTCT knowledge, safer conception, family planning and dual method sexual barrier use. Intervention participants will attend 3 prenatal weekly 2 hour gender-specific (male or female, 5-7 participants) group sessions followed by 3 monthly couples or individual (women-only) counseling sessions (1 prenatal, 2 postpartum) led by study-trained clinic staff (e.g., nurses, HCT counselors) plus SOC. The intervention strategies are in accordance with the SA PMTCT 2010 Guidelines (SA, 2009), PMTCT Expert Panel (2010) and Guidance for Healthcare Providers (Engender Health, 2008).

**Group sessions**. Sessions address prevention of vertical transmission, the importance of adherence to PMTCT and medication use, prevention of unintended pregnancy, HIV testing of family members and prevention of sexual transmission of HIV. Participants receive cognitive behavioral skill training addressing the key components of each session, e.g., how cognitions relate to anticipated outcomes and thereby predict behavioral change. Group members are encouraged to problem solve, providing supportive feedback and peer mentorship on session topics. Session topics for men’s and women’s groups are comparable, but place different emphasis on gender relevant topics, e.g., women focus on PMTCT protocol and medication adherence; men focus on HIV testing, alcohol and drug use. Participants role play communication strategies and complete communication and sexual negotiation homework using strategies practiced in the group sessions. Emphasis is placed on the sequence of communication, disclosure and avoiding IPV, e.g., women focus on prevention of IPV and conflict resolution, while men focus on anger management and prevention of IPV; both identify the antecedents of conflict and IPV as a method of prevention. Women establish a “safety plan” for responding to the threat of IPV, and are encouraged to address concerns about personal safety with clinic staff during study participation. Intervention information is presented with opportunity to practice new communication strategies and receive feedback (e.g., discussion of the pros and cons of disclosure, sharing experiences on partner’s responses). [N.B.: Men’s sessions do not address their partner’s HIV status.]

**Group intervention sessions 1-3.**

**Session 1**: HIV informational review, HCT (men’s focus), PMTCT, infant feeding, ARV medication initiation/management (women’s focus), communication strategies for sensitive topics, avoiding IPV, safer sex with male & female condoms. Cognitive/behavioral (CB) skill training heightens participant awareness of their reactions to PMTCT, condom use, communication. Condom promotion introduces “novelty” condoms (colors, flavors, textures, sensations, sizes) to increase condom acceptability through “eroticization.” Participants receive a week’s supply of male “novelty” & female condoms.

**Session 2**: PMTCT discussion, HIV stigma, disclosure, family planning & ART (women’s focus), HCT, alcohol and drugs, communication (men’s focus), prevention of IPV. CB skills are applied to improving communication techniques. CB skills are used to address anxiety regarding HIV status disclosure and safer conception negotiation, including family planning post-partum.

**Session 3**: PMTCT, HAART, HCT discussion, family planning, safer conception, dual barrier methods & disclosure (women’s focus), conflict resolution & anger management (men’s focus), communication & reducing/avoiding IPV, cognitive behavioral skill training, and importance of health facility delivery and PMTCT medications at birth. CB exercises and role plays use participant experiences in problem solving, and participants are guided in applying cognitive restructuring skills to conflict resolution, communication, safer sex, and HIV disclosure. Participants are provided with a week’s supply of male and female condoms.

**Counseling sessions.** Three, structured one-hour sessions are led by study-trained CHC staff with couples or individuals (women-only). The first session is antenatal, two additional sessions occur 6 weeks and 3 months post-natally. Information concerns adherence to the PMTCT protocol, e.g., infant feeding practices and medication, and reproductive decision making, e.g., fertility planning, safer conception practices. Each session enhances Motivation to adhere to the PMTCT protocol, i.e., the necessity to adhere to the protocol throughout the infant’s first 6 months of life, or longer, as determined by the child’s health status. Each session also addresses reducing risk Behavior related to unintended pregnancy, acquisition of STDs and prevention of STD/HIV transmission to partners, and use of dual methods of protection (i.e., consistent use of condoms along with another safe contraceptive method) regardless of partner serostatus. [N.B.: If disclosure has not occurred and is not planned counseling sessions will focus on family planning and dual protection methods, infant feeding practices and safer conception and will not compromise participants’ confidentiality.]

**Counseling sessions 1-3.**

**Session 1**: **Week 32** **Antenatal .** Review of PMTCT protocol (adherence to medication, infant feeding practices). Importance of health facility delivery & NVP at delivery. Reproductive decision making: discussion of fertility intentions & safer conception practices. **Session 2**: **6 weeks Post-natal.** Review of PMTCT protocol (medication, infant feeding). Discussion of safer conception practices and family planning using dual barrier methods. **Session 3: 3 months** **Post-natal.** Review of PMTCT protocol (medication, infant feeding) and review of use of safer conception and risk reduction practices.

**Control & PMTCT Standard of Care.** Participants will receive PMTCT SOC plus a time-equivalent, group-administered video presentation on childhood disease prevention (e.g., measles, diarreal management, dysentery/dehydration, immunizations/vaccinations) in 3 group sessions, followed by 3 couple or individual women sessions on childhood disease prevention.

**Implementation, Sustainability & Generalizability.** If successful in reducing MTCT, the study investigators will meet with the District, Regional and Provincial health officials to promote the translation of study findings into health policy recommendations. The investigators will promote program implementation and scale-up at other health centers within Mpumalanga and other South African provinces (e.g., Kwa-Zulu Natal) with notably elevated rates of MTCT. The intervention manual, train the trainer model for clinic staff and QC materials provide a framework for implementation and sustainability for CHC staff as both implementers and trainers. This strategy will enable rapid scale-up into other ANC/CHC/hospital venues at District, Provincial and National levels. Current studies support the sustainability of skill building programs for CHC staff to achieve expanded scope of practice (e.g., Uwimana et al., 2012a; Peltzer et al., 2009; 2010). The DOH now employs at least 5 paid, trained, certified community health workers per clinic rather than relying on volunteers, who will be available to implement the intervention on a large scale (e.g., Nxumalo et al., 2013; Ndou et al., 2013; Uwimana et al.,2012b).

**Challenges & Solutions**. Ongoing project staff/CHC meetings will be used to assess and respond to challenges as they arise. Challenges and solutions for implementation are described, and include 1) attainment of recruitment goals: if needed, additional sites have been identified for recruitment in neighboring Ehlanzeni District, 2) retention in care: our previous pilot study achieved 95% retention over 12 months utilizing a protocol of active participant contact and updates to participant locator information at each visit which will be utilized in the proposed study, 3) non-HIV disclosure prior to couples counseling session: a protocol will be established prior to counseling on maintaining HIV confidentiality, 4) IPV risk: the pilot study established a protocol to respond to IPV and threats of violence that will be utilized in the current study; [N.B.: Pilot study found a reduction in IPV and negative communication in the experimental condition], 5) contextual and health system challenges: study investigators and staff will engage local stakeholders, community advisory boards, leadership of CHCs and Provincial MOH in the planning and implementation process, 6) clinic site staff turnover and space limitations: clinics and CHC staff will be supported in the designation of devoted space for groups, and the identification of qualified staff to participate and the use of the train the trainer model to provide additional trained staff to anticipate potential staff turnover.

**Analytic Plan**

**Statistical Analyses.** The study tests an intervention to reduce vertical transmission by enhanced PMTCT uptake using an intent to treat model. A cluster randomized design will be used, with 12 clinics stratified by size and randomly assigned as 6 intervention and 6 control clinics with 60 women (Phase 1) and 60 couples (Phase 2) per clinic. Preliminary analyses will include descriptive statistics, e.g., means, standard deviations, frequencies and percentages as well as t-tests, chi-square tests, Pearson’s correlations of factors associated with sexual risk behavior, medication adherence, retention in care and vertical transmission. Factors considered as potential confounders are those observed to have moderate associations with outcome variables in preliminary tests of association, using a conservative α of 0.20 for significance testing. These variables will be controlled in analyses as appropriate and assessed independently, e.g., male partner, male partner serostatus, HIV disclosure, IPV, communication style. Prior to analyses, appropriate variable transformations will be applied to outcome variables in order to satisfy distributional assumptions. SAS (SAS Institute, Inc., Cary, NC) and SPSS (Statistical Packages for Social Sciences, IBM) will be used for all analyses using the 0.05 level to determine statistical significance. These analyses have random terms that account for variation caused by treating subjects in a group and in a clinic (individuals nested within cohorts; cohorts are clustered in clinics); these are random terms in the analytic models. The following hypotheses will be adjusted for the clustering effect.

**Specific Aim 1**: to increase uptake and adherence to ante-, peri- and post-natal PMTCT protocols by HIV positive pregnant women through the implementation of a comprehensive, evidence-based risk reduction, medication adherence and PMTCT intervention.

**Hypothesis 1.1:** Within each Phase, Experimental condition mothers will be significantly more likely to adhere to PMTCT protocol medications as prescribed compared to Control condition mothers at 32 weeks pregnant, and 6 and 12 months postpartum, and provide them to infants up to 6 weeks.

**Hypothesis 1.2:** Within each Phase, infants born to Experimental condition mothers will be less likely to test HIV+ at 6 weeks and 12 months of age compared to those born to Control condition mothers.

**Specific Aim 2**: to retain women and infants in post-natal care to ensure adherence to PMTCT safe infant feeding protocols (SA DOH, 2010).

**Hypothesis 2:** Within each Phase, experimental condition mothers will be more likely to continue PMTCT safe infant feeding at 6 months and more likely to be retained in care at 6 and 12 months post-partum compared to control condition mothers.

**Specific Aim 3**: to improve sexual and reproductive decision making and safer conception practices.

**Hypothesis 3:** Within each Phase, Experimental condition mothers will decrease unsafe sexual practices and/or increase and maintain the use of safer conception practices (e.g., dual barrier methods) at 32 weeks pregnant and 6 and 12 months post-partum compared to Control condition mothers. This will be accomplished with no increase in intimate partner violence.

**Specific Aim 4**: to assess the impact of male engagement on PMTCT uptake (medication adherence, feeding practices, clinic attendance), safer sex and family planning practices.

**Hypothesis 4:** Within the Experimental condition, mothers in Phase 2 (couples arm) will a) be more likely to take PMTCT medications at 32 weeks pregnant and 6 and 12 months postpartum, and provide medications to their infants up to 6 weeks; b) be more likely to continue safe infant feeding and be retained in care at 6 and 12 months; and c) be more likely to increase safer sex and/or safer conception practices at 32 weeks pregnant, and 6 and 12 months in comparison with mothers in the Experimental condition Phase 1 (women-only arm).

**Analytic Approach**: In order to test the impact of the experimental condition within each Phase on the outcomes of Hypotheses 1.1, 1.2, 2 & 3, separate analyses will be conducted for the women-only Phase and the couples Phase. A series of general linear mixed models will be conducted to perform regressions with adherence, infant HIV serostatus, safe infant feeding, retention in care, safer sex, family planning practices as the outcomes and clinic, condition status (intervention vs. control), time, and the interaction of time and condition status as the fixed predictor of interest. The random effects will be persons nested within cohorts and cohorts nested within clinics. A heterogeneous autocorrelated covariance matrix will be used to represent the correlated data structure. A secondary analysis will test for moderating effects such as male partner, HIV serostatus disclosure, self-efficacy for disclosure, and male partner serostatus. Significant moderators will be kept in the final model. Planned comparisons will be made between groups at each time and between times within each condition or Phase. For Hypothesis 4, Phase 1 and Phase 2 will be compared using the same type of analysis on the intervention condition only, and include as fixed predictors Phase, time, and Phase x time interaction, utilizing the same random effect and covariance structure.

In order to test the relative contribution of ART adherence, safe infant feeding and retention in care on Infant HIV PCR outcome at 12 months, additional analyses for Hypothesis 1.2 will be conducted with a general linear mixed model, performing a logistic regression in which infant HIV PCR at 12 months will be the outcome, and ART adherence, safe infant feeding and retention in care will be the fixed predictors of interest at 12 months with a random effect of person within cluster.

Based on our experience with this population (see Preliminary Studies), we expect to have complete data for baseline and post intervention. Retention rates for follow-up at 6 and 12 months are expected to be 95% at each time point. Therefore, we assume any missing data is random and non-informative. This type of missing data is permissible for mixed model analyses. Missing data will be reviewed for systematic trends within condition and controlled for, as appropriate.

**Power Analysis.** The power calculations for Hypothesis 1.1 used a pooled analysis of adherence studies that indicated a pooled estimate of 73.5% (95% confidence interval [CI] 69.3-77.5%, *I^2^*=97.7%) of pregnant women had adequate (>=80%) ART adherence. The pooled proportion of women with adequate adherence levels was higher during the ante-natal (75.7%, 95% CI 71.5-79.7%) than during the post-partum period (53.0% to 95%; 32.8% to 72.7%) (p=0.005). Calculations for Hypothesis 2 are based on our pilot study data and local assessments (Ukpe et al., 2009; Ladzani et al., 2011), which identified 30-50% of sero-positive mothers providing mixed feeding post-natally. Our estimated sample size is based on infant serostatus at 6 weeks and 12 months. Averaged clinic data indicate ~13% of infants will be seropositive at 6 weeks and an additional 13% will be seropositive at 12 months of age. Using an HIV PCR rate of 13% at 6 weeks in the control arm, a power analysis for Hypotheses 1.3 and 3 indicated that 6 sites per group (6 experimental, 6 control) with an unadjusted sample size of 564 infants per phase would provide 80% power to detect a significant difference between conditions assuming reduction to 4% in the intervention condition and intracluster correlation coefficients (ICC) up to 0.02 (depending on the two rates) with a two tailed test at the 0.05 level (Klar & Donner, 2001). This calculation assumes Infant HIV PCR rates of 13% in the control arm and rates of 4% in the experimental arm at 6 weeks.

**Sample size, missing data and attrition**. Missing data will be reviewed for systematic trends within condition and controlled for if appropriate. The sample size of 720 pregnant women per phase was based on our experience in our current PMTCT study, from which we anticipate a 16% miscarriage and infant death and a 5% attrition rate over 12 months (n = 156 lost; n = 564 retained).

## Laboratory Methods

### Specimen tracking

Batches of specimens will be sent to a laboratory at the University of Cape Town and will be tracked through waybill procedures. Specimens and specimen tracking sheets with the DBS bar-code will be sent to the laboratory in transparent zipper lock bags containing desiccant. Consecutively numbered laboratory bar-codes will be assigned to the specimens as they are received by the laboratory. The specimen bar-codes will by matched to the bar-codes on the laboratory tracking sheets. The specimen bar-code number will also be scanned or typed into an Excel spreadsheet. The Guthrie cards will also be labelled with the laboratory bar-code number. Laboratory managers will perform a second quality control (matching bar-codes to tracking sheets and examining specimen quality) and will sign-off the tracking sheets for laboratory processing.

Detection of antiretroviral drugs

The presence of antiretroviral drugs (ARVs) in HIV positive DBS samples will be confirmed by means of High

Performance Liquid Chromatography (HPLC) coupled to Tandem Mass Spectrometry. Qualitative detection of Lopinavir, Ritonavir, Nevirapine, Efavirenz, Indinavir, Saquinavir, Zidovudine, Lamivudine and Stavudine in DBS samples wll be carried out by a validated method using minor modifications of the method used by Koal et al. (2005). Antiretroviral drugs are extracted from the DBS samples with 80% methanol and 20% 0.2M Zinc

Sulphate containing an internal standard. HPLC is carried out on a Phenomonex Fusion RP column (56264mm) using a methanol/10 mM ammonium acetate gradient t to effect elution. Detection of antiretroviral drugs is carried out using an Applied Biosystems API 4000 tandem mass spectrometer in the multiple reaction monitoring (MRM) detection mode for each drug using appropriate MRM transitions. Blank and quality control cutoff samples are included with each run. The limit of detection for each drug will be set at 50 ng/ml, a sensitivity set point which is normally applied for quantitative monitoring of drug levels in the blood. Values detected above this limit were considered as positive and those below as negative (Rehle et al. 2010).

**HUMAN SUBJECTS**

Ethical approval will be obtained from the University of Miami Miller School of Medicine (UM) and the Human Subjects Research Council (HSRC) Ethics Committee and the Health Department of South Africa, Mpumalanga Province, prior to study onset.

**1. Sources of Research Material**

Psychosocial and behavioral data will be collected over a total of four assessment time points [1) baseline, 2) post-intervention (32 weeks pregnant), and postpartum 3) 6-months and 4) 12-months post-delivery]. All psychosocial assessments will be conducted using audio computer assisted self interview (ACASI) technology.

Where possible, to reduce participant burden arising from repeated testing, standard of care HIV counseling and testing outcomes of mother, father and infant will be obtained by clinic record extraction. Study biological assessments will be conducted in the following sequence, which includes assessments conducted within the existing standard of care (SOC): 1) Women only: HIV baseline status (SOC), ARV uptake by dried blood spot (DBS) pre-delivery at 32 weeks pregnant (study); Infant only: ARV uptake by DBS (study) and HIV status by PCR at 6 weeks (SOC) and 12 months (study); Men only: HIV status across the duration of the study, if tested (SOC).

**2. Participants**

A total of 720 HIV positive pregnant individual women and 720 HIV positive pregnant women and their male partners (N = 2160) aged 18 and older will be recruited for this study from 12 Antenatal Clinics (ANCs) in Mpumalanga Province.

Women will participate in one of the following conditions, determined by clinic randomization (control, experimental) and time of study entry (Phase 1:1st 720 women & Phase 2: 2nd 720 women and male partners). The first cohort of 720 women (Women-only, Phase 1) will be recruited and subsequently, an additional cohort of 720 women with their male partners (Couples, Phase 2) will be recruited.

Each woman may only enroll for one pregnancy.

A total of 1440 infants will participate in this study. Infants will be born to mothers participating. Mothers will be asked to provide consent for their newborn infants to provide 2 blood samples; dried blood spot (DBS) at 6 weeks to assess for antiretroviral medications (ARVs), and an HIV test at 12 months.

**Language.** All study staff will be conversant in English, Ndebele, Zulu and Swati. All informed consent and assessment materials will be translated into IsiZulu, SiSwati or Ndebele.

**3. Study Procedures - Recruitment.**

**Referral.** Women candidates will be referred by antenatal clinic (ANC) PMTCT/HIV Counseling & Testing (HCT) staff to recruiters/assessors following HCT, which is provided as the Standard of Care (SOC) upon entry to care in the ANC. All women will have tested HIV seropositive at ANC entry or prior to ANC entry; clinic records will be used to confirm serostatus. Per South African PMTCT Protocol, all women receive pre- and post-HIV counseling and testing and, if HIV+, referral for CD4 assessment and ART. Following a positive HIV test or if clinic records indicate a woman has previously tested HIV seropositive, women will be informed about the study and referred to study staff for more information on the study.

**HIV Status Confidentiality.** Confidentiality of the serostatus of both couple members will be ensured throughout the study on all documents, questionnaires and during all sessions. Per the standard of care, male partners will be encouraged, but not required, to undergo HCT.

**Study review.** Recruiters will meet with interested women to present the study. During the information session, the recruiter /assessor will describe the nature of the study to prospective participants, assure them of the confidentiality of all information including their serostatus, and inform them that they may refuse participation at any time. Women will be advised of the eligibility criteria for enrollment (see below), and the study objectives and procedures will be thoroughly explained by the study staff member.

**Inclusion/Exclusion Criteria.** The nature of the intervention requires the participants to be HIV seropositive pregnant women with a male partner, 24-32 weeks pregnant (typical time of entry into ANC care), 18 years of age or older and willing to participate in the Protect Your Family (“Vikela Umndeni”) program. Women not having a male partner are not eligible to participate.

Male partners. For the purposes of this study, primary male partners are defined as 1) husband, 2) current baby’s father, or 3) current sexual partners for the 6 past months.

**Eligibility.** Participants will be HIV seropositive pregnant women with a male partner, 24-32 weeks pregnant (typical time of entry into ANC care), 18 years of age or older and willing to participate in the Protect Your Family (“Vikela Umndeni”) program**.**

**Women declining to participate.** In order to assess potential systematic recruiting bias, women declining to participate will be asked to indicate the reason for declining using a brief open-ended screening tool (e.g., inconvenient, no interest, partner unwilling, concerns about IPV, confidentiality). No unique identifying information will be collected from female participants at this stage.

**Recruitment.**

**Phase 1**: Women-only phase, women will be asked to provide Informed Consent and complete a baseline study assessment with ACASI.

**Phase II:** Couples phase, male partners will be invited to attend by their female partners following initial study briefing with the study recruiter.

**Phase II Screen.** Participants in Phase 2 (couples) will complete a pre-enrollment screening interview to confirm couples status with their partner, during which time both participants will be asked three of a rotating series of questions designed to confirm partners, e.g., Which side of the bed do you sleep on? What color is your blanket? These screening questions have been successfully used in our previous studies in Zambia and South Africa. If couple members cannot correctly answer the screening questions, they are thanked for coming and not eligible to participate.

Phase II couples will provide Informed Consent individually, and couples will individually complete a baseline study assessment with ACASI.

**4. Study Procedures – Informed Consent:**

The informed consent will be reviewed in detail and read aloud to the prospective participant in their primary or selected language; the consent is then reviewed section by section for comprehension. Study candidates are asked to recount the essential content of the informed consent back to the staff member using a consent comprehension test, and agreeing to the terms of the consent before signing the form. Any items of confusion are clarified by the study recruiter. Each study candidate will be asked to sign or provide their mark on the form after reading or having the contents read and explained, and if agreeing to its terms, and the signature of a witness will be obtained.

The following questions will be used to evaluate comprehension of Informed Consent.

Purpose: Can you tell me in your own words what this project is all about?

Procedure: Can you tell me how many times you are expected to come to the study site?

Can you tell me how long you will be coming?

Risks: Can you tell me if there are any risks in participating in the study?

Compensation: Can you tell me if there is any compensation for coming?

Confidentiality: Can you tell me how your records will be kept safe?

Summary: Do you have any questions or concerns about the study?

**Confidentiality.** Each participant will receive a card with a code number on it upon entering the study and will be instructed to bring this card to all visits. This number will identify all participant files, with all other identifiers removed. Access to the computer data files will be restricted by password codes. At each assessment visit, the participant will reveal his/her code number and name so locator and health data files can be updated. The list translating participant number to identifying information will be maintained in a secure locked file in the HSRC office in Pretoria. Additionally, in order to be able to track participants across the longitudinal period of the study, we will keep a separate record of each participant’s address and contact person information and the participant number. This record will indicate whether or not a participant has completed an assessment but will not include any assessment or intervention performance data. Participants will be made explicitly aware at the time of the informed consent of the nature of the two separate records that will be kept for them. Intervention sessions will be audio-taped for quality control; tapes will be stored in a locked cabinet at the PI’s HSRC offices, and destroyed within 5 years of completion of the study. N.B. Study Informed Consent protocol will ensure that participant confidentiality is maintained regarding HIV serostatus. ACASI and study session materials will also be designed to ensure confidentiality of all information collected, including HIV serostatus of both couple members.

**5. Study Procedures – Intervention & Control Conditions**

**Study Randomization.** Twelve CHCs will be stratified by size and randomly assigned as experimental or control sites. Participants at both the experimental and control site conditions will receive baseline, post intervention, 6 and 12 month post-delivery assessments.


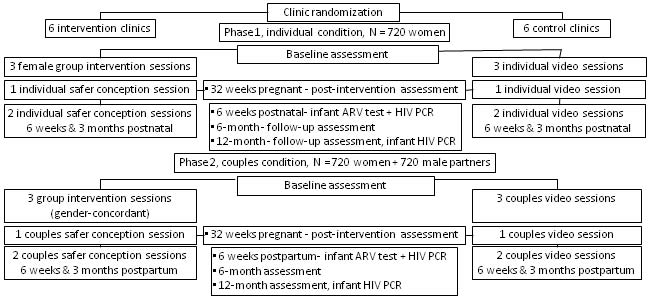


Women or couples participation:

• Phase 1: women attend a gender-specific (women-only) intervention or control group.

• Phase 2: women and their male partners attend gender concordant intervention groups (women-only group & men-only group) or gender concordant control groups.

**All study participants will attend a) 3 gender-concordant group sessions and b) 3 individual or couple sessions, c) complete 4 ACASI assessments and d) their infants will provide 2 blood samples.**

**Intervention and Control Conditions.**

**Behavioral Intervention.** “**Vikela Umndeni”(Protect Your Family)** intervention participants will attend 3 prenatal weekly 2 hour gender-specific group sessions followed by 3 monthly couples or individual counseling sessions (1 prenatal, 2 postpartum) led by study-trained clinic staff members (e.g., nurses, VCT counselors). The strategies in the intervention are in accordance with the SA PMTCT 2010 Guidelines (SA, 2009), PMTCT Expert Panel (2010) and Guidance for Healthcare Providers (Engender Health, 2008).

“**Vikela Umndeni” group intervention** is a manualized, closed, structured male or female group (5-7 participants) behavioral risk reduction program targeting HIV, stigma, disclosure, communication, IPV, PMTCT, safer conception and family planning, and dual method sexual barrier use. In each session, the program addresses prevention of vertical transmission, the importance of adherence to PMTCT and medication use, prevention of unintended pregnancy, HIV testing of family members and prevention of sexual transmission of HIV. Participants receive cognitive behavioral skill training addressing the key components of each session, e.g., how cognitions relate to anticipated outcomes and thereby predict behavioral change. Group members are encouraged to problem solve, providing supportive feedback and peer mentorship on session topics. Session topics for men’s and women’s groups are comparable, but place different emphasis on gender relevant topics, e.g., women focus on PMTCT protocol and medication adherence; men focus on HIV testing, alcohol and drug use. Participants role play communication strategies and complete communication and sexual negotiation homework using strategies practiced in the group sessions. Emphasis is placed on the sequence of communication, disclosure and avoiding IPV, e.g., women focus on prevention of IPV and conflict resolution, while men focus on anger management and prevention of IPV; both identify the antecedents of conflict and IPV as a method of prevention. Women establish a “safety plan” for responding to the threat of IPV, and are encouraged to address concerns about personal safety with clinic staff during study participation. Intervention information is presented with opportunity to practice new communication strategies and receive feedback (e.g., discussion of the pros and cons of disclosure, sharing experiences on partner’s responses).

**Group intervention sessions 1-3.**

**Session 1**: HIV informational review, HCT (men’s focus), PMTCT, infant feeding, ARV medication initiation/management (women’s focus), communication strategies for sensitive topics, avoiding IPV, safer sex with male & female condoms. Cognitive/behavioral (CB) skill training heightens participant awareness of their reactions to PMTCT, condom use, communication. Condom promotion introduces “novelty” condoms (colors, flavors, textures, sensations, sizes) to increase condom acceptability through “eroticization.” Participants receive a week’s supply of male “novelty” & female condoms.

**Session 2**: PMTCT discussion, HIV stigma, disclosure, family planning & ART (women’s focus), HCT, alcohol and drugs, communication (men’s focus), prevention of IPV. CB skills are applied to improving communication techniques. CB skills are used to address anxiety regarding HIV status disclosure and safer conception negotiation, including family planning post-partum.

**Session 3**: PMTCT, HAART, HCT discussion, family planning, safer conception, dual barrier methods & disclosure (women’s focus), conflict resolution & anger management (men’s focus), communication & reducing/avoiding IPV, cognitive behavioral skill training, and importance of health facility delivery and PMTCT medications at birth. CB exercises and role plays use participant experiences in problem solving, and participants are guided in applying cognitive restructuring skills to conflict resolution, communication, safer sex, and HIV disclosure. Participants are provided with a week’s supply of male and female condoms.

**“Vikela Umndeni” counseling** is 3 structured one-hour sessions led by trained study CHC staff with couples or women-only. The first session is antenatal, two additional sessions occur 6 weeks and 2 months post-natally. Sessions address adherence to the PMTCT protocol, e.g., infant feeding practices and medication, and reproductive decision making, e.g., fertility planning, safer conception practices. Each session reviews the current adherence to the PMTCT protocol, i.e., the necessity to adhere to the protocol throughout the infant’s first 6 months of life, or longer, as determined by the child’s health status. Each session also addresses risk reduction related to unintended pregnancy, acquisition of STDs and prevention of STD/HIV transmission to partners, and reviews dual methods of protection (i.e., consistent use of condoms along with another safe contraceptive method) regardless of partner serostatus. *If disclosure has not occurred and is not planned counseling sessions will focus on family planning and dual protection methods, infant feeding practices and safer conception and will not compromise participants’ confidentiality.*

**Pregnancy safer conception counseling sessions 1-3.**

**Session 1**: **Week 32** **Antenatal .** Review of PMTCT protocol (adherence to medication, infant feeding practices). Importance of health facility delivery & NVP at delivery. Reproductive decision making: discussion of fertility intentions & safer conception practices. **Session 2**: **6 weeks Post-natal.** Review of PMTCT protocol (medication, infant feeding). Discussion of safer conception practices and family planning using dual barrier methods. **Session 3: 3 months** **Post-natal.** Review of PMTCT protocol (medication, infant feeding) and review of use of safer conception and risk reduction practices.

**Control & PMTCT Standard of Care.** Participants will receive PMTCT plus a time-equivalent, group-administered video presentation on childhood disease prevention (e.g., measles, diarreal management, dysentery/dehydration, immunizations/vaccinations) in 3 group sessions, followed by 3 couple or individual women sessions on childhood disease prevention.

**6. Study Procedures – Compensation, Retention & Attrition**

**Compensation.** Given the time demands of this study and the personal nature of disclosures required participants will be offered South African Rand 50 (~US$ 6) per assessment visit for time and transport. Snacks will be provided during each group and individual session in both conditions and both Phases.

**Participant Retention & Attrition**.

**Retention.** Our pilot study conducted in Mpumalanga ANCs achieved study retention rates of 95% - 100% over the course of the study (4 months pregnant to 3 months post-delivery, respectively). These research programs have been imbedded in the ANC PMTCT service component, which encourages participant attendance at assessment and intervention sessions. Study staff attempt to locate participants who miss study assessments for a two-week period following failure to present for the appointment. If, over a period of 2 weeks of continued attempts, staff are unable to reach a participant to re-schedule an assessment, staff attempt to reach the contact person that participants provided at study entry to learn of the whereabouts of the participant. Home visits are conducted if all efforts to contact participants are unsuccessful. Staff ensure maintenance of participant confidentiality during all contacts.

**Attrition.** Participants are considered lost to follow up or no longer in the study if 1) they cannot be found following a concerted attempt for location (see above), or 2) participants notify staff that they are no longer interested in participating in the study.

**7. Potential Risks/Benefits Ratio**

PMTCT HIV testing for mother and infant is administered in accordance with the current South Africa PMTCT Protocol as a clinical service at the CHC. HIV testing for men and women is not offered as a component of this study, and is administered as a clinical service at the community health center (CHC); informed consent for HIV testing is obtained by the CHC staff prior to provision of services.

Biological data will be collected by finger or heel stick using a lancet to obtain a dried blood spot to assess anti-retroviral use from the HIV positive mother pre-delivery (32 weeks) and the infant at 6 weeks. There may be some minor discomfort from the finger or heel prick using a small pin or lancet when collecting a few drops of blood. Infants will also be tested to assess HIV serostatus at 12 months. In the proposed study, potential risks also include those associated with use of sexual barrier products, such as an allergic reaction to latex. Should any side effects occur, participants will be directed to discontinue use of specific products and referred for appropriate medical treatment as needed.

Participants should incur no other appreciable physical risks. Through participation in this study they may undergo psychological or physical discomfort at some times, including embarrassment, discomfort, or distress during groups discussing sexual activity, violence, alcohol use, and HIV. For example, during the intervention, extensive discussion regarding HIV disclosure may lead some participants to experience mild, transient anxiety. In addition, during the intervention, participants may be asked to participate in role-playing sessions (e.g., disclosure) that may lead some participants to experience mild, transient anxiety.

It is also possible that during the course of our contact with participants we may become aware of problems that fall outside of the scope of the proposed research but require intervention. Participants will be advised that there are certain ethical limits to confidentiality. Should any participant disclose that he/she is experiencing suicidal ideation, he/she will be immediately evaluated by a qualified physician or psychologist and actions may be taken to protect others and him or her. If a participant tells the researcher that he or she plans to in any way physically or sexually harm an identifiable person including a spouse or partner, it will be required that the researchers take steps to intervene. If the participant tells the researcher that he or she believes an identifiable person is going to physically or sexually harm him or her, steps will be taken to protect him or her.

**Special considerations regarding the potential for intimate partner violence.** Should participants experience extreme acute or persisting affective reactions at any point during the study period due to the risk or anticipation of intimate partner violence (IPV; see below for details), they will be referred to medical and/or human services professionals associated with the CHC for care. Following this evaluation, if necessary, the participant will be accompanied to the referral hospital for further assessment and/or hospitalization, or, if not in immediate danger of harming her/himself, will be referred for outpatient treatment. [N.B.: IPV has been a major concern in our HIV risk reduction studies over the past 15 years. We are pleased to report that either no increase or a reduction in IPV has been observed post-intervention, despite the sensitivities of issues such as disclosure of status or use of sexual barriers among couples. We believe post-intervention improvement in couple communication and coping skills as well as anger management/ assertiveness training have contributed to these reductions in IPV, even in venues with high rates of IPV such as South Africa (see Preliminary Studies)].

Potential risks to participants in this study also include negative consequences that may be suffered if confidentiality of information obtained in the study (including subject identity as a research participant) were breached. A number of steps will be taken to protect the confidentiality of participant data and identity. Study staff will attend training sessions by the study investigators and receive ongoing supervision in areas related to ethical conduct, confidentiality protection, and other topics of human participant protection. We will ensure that the recruiters are trained to explain the purpose of the study to potential respondents, obtain informed consent, and inform respondents about their rights and benefits in a factual and neutral way without coercion to participate. We will also ensure that our interviewers inform the potential respondents about the confidentiality measures put in place to protect their privacy. The demographic and psychosocial ACASI assessments are confidential and conducted in a private office area inside each clinic and all research data obtained from participants will be labeled as a computer file with a code number and not the participant’s name. Only the number will appear on measures, data records, and computer files. A master list that includes participant identifying information and identifying codes will be kept in a locked file in a locked office at the HSRC.

**Procedures related to Intimate Partner Violence (IPV).** Assessment of intimate partner violence (IPV) will be included in the study assessment at each major study time point. In the event of a report of IPV, the CHC protocol for responding to reported IPV is 1) interview with offending partner (usually the husband), 2) medical examination of victim spouse and possible inpatient admission or 3) referral to the alternative community shelter for mother and children, provision of 4) medical treatment and counseling support. Based on the results of the medical examination, 5) a police report is also submitted by CHC staff and 6) police will then interview the domestic partner (husband). In the case of reported abuse of children, children are 1) assessed and as appropriate, 2) placed in the protective care.

Following a report of IPV, all subsequent sessions and assessments will be conducted independently; no partners will attend sessions or assessments at coinciding times. In the event that both partners request to reunite, they will begin assessments at coinciding or overlapping times, if they prefer.

All staff will be trained in requirements of confidentiality and anonymity. ACASI will be used for all assessments in order to ensure confidentiality between study staff and participants. Finally, additional safeguards will be established to provide support to any participants wanting assistance in disclosing their status to their partners; VCT staff will be utilized to conduct disclosure as they are specially trained to facilitate disclosure within couples. Staff will also provide support and referral to any participants at risk of intimate partner violence (IPV); a protocol has been established for referral of participants who identify themselves as at risk of IPV. The protocol was used in our previous study and includes, staff training on prevention of IPV, planning for safety, removed to places of safety, reporting IPV to health care providers and police, and obtaining protection orders. Staff will receive additional training through POWA (People Opposing Women Abuse), an NGO throughout South Africa) on techniques to ensure safe outcomes in situations in which IPV may arise following disclosure or other conflict.

In the event that the study enrolls a woman who has not disclosed her status WHO WISHES TO DO SO, the study staff member will accompany the women to the HIV VCT Counsellor (see above). The VCT Counsellor is specially trained in facilitating disclosure and will assist in the disclosure process with the current partner. Following disclosure, or at any time during participation in the study:

In the event that the woman (or man) discloses that they are at risk of IPV, the study staff member will review with the participant the techniques for prevention of IPV, planning for safety, removal to safe houses, reporting IPV to health care and police, and procurement of restraining orders (stay away), and provided with the phone numbers for the POWA hotline (011 642 4345/6) and the police. Study staff members will be specially trained in these procedures.

**Benefits.** Potential benefits to participants include information and experiences that optimize PMTCT protocol adherence, family planning, safer reproductive strategies, safe infant feeding practices and sexual health. Participants should benefit from training in anxiety reduction, coping skills, and communication, as well as non-specific effects of membership in a supportive group under the guidance of trained group leaders.

Time needed for assessments is approximately 60 minutes and intervention sessions are approximately 1.5-2 hours. There are no costs to participants, and financial compensation is provided at each assessment.

**8. Biohazards.**

There are no biohazard risks to study participants.

Data Safety and Monitoring Plan

A Data Safety Monitoring Board (DSMB) will be established prior to study onset. The Board will include two members, one from the HSRC in SA and one from the University of Miami in the US, neither of whom will be directly connected with the study. A review will be conducted at study midpoint; board members will review the data to determine: 1) if there are any unexpected systematic variations in health status attributable to either study condition which may be negatively affecting the health of the participants; and 2) whether the experimental condition (or control) are demonstrating such clear-cut positive effects that continuation of the trial (i.e., withholding the effective treatment from half of the participants) would be considered unethical. Prior to the initiation of the study, the Project Executive Committee will appoint members for the DSMB; the DSMB and Project Executive Committee will develop stopping rules to ensure participant safety.

Project Leadership Plan

**Leadership Plan - Multiple PIs**

Roles of the PIs: The following management structure is already in place and will be maintained at both the Miami and South Africa sites. Two project management teams (Miami: Jones, Weiss, Fischl; South Africa: Peltzer, Mwisongo) ensure appropriate coordination, consistency and quality control in implementing the research protocol across the two sites. The PIs will be responsible for the administration of the study at their respective sites, including Institutional Review / Ethical Review and approval, implementation of protocol and procedures, and oversight of issues related to human subject protections.

Fiscal and management coordination: PIs are jointly responsible for scientific direction and for fiscal management at their respective sites. Dr. Jones will be responsible for the overall fiscal management as the University of Miami is the primary grantee.

Process for Decision Making: Drs. Jones, Peltzer, Weiss, Mwisongo, Fischl, Arheart and the South Africa statistician comprise the Project Executive Committee and are responsible for procedural issues and program management.

The Project Executive Committee, chaired by Dr. Jones, will provide overall policy development, guidance and quality assurance for the study. This committee will be responsible for implementation of the aims of the study and maintaining communication across both sites. Policy guidelines for the project and quality assurance include standards for staff training, assessment, treatment, quality control and maintenance procedures. Assessment and Intervention Manuals and the original Manual of Operations will be created in Miami and adapted as needed to the South African context through consensus by both teams.

The Project Steering Committee, chaired by Dr. Peltzer, will be the operational arm of the study, responsible for specific management (administration) and design issues that may impinge on study conduct and/or outcome and operating procedures. The objective of the Committee will be to standardize and maintain integrity of all procedures for recruitment, intake, assessment, treatment, maintenance and follow-up. Regular email communication and monthly conference calls over the course of the project will maintain a regular system of reporting on issues that may affect the conduct of the study.

Data sharing and Communication: Data will be collected at the South Africa site, and reviewed for quality control at the US site. Both sites will have access to the final dataset for analyses.

Communication will be maintained by scheduled monthly calls, supplemented by email and FAX. The PI and Co-PI will travel to South Africa at the onset of the study to finalize all guidelines and protocols and train staff Drs. Jones and Weiss will travel to the South Africa site twice yearly for the duration of the study (see Budget Justification).

Publication and intellectual property: All publications will be reviewed and shared between sites, and the final dataset, with no unique identifiers, will be made available as a shared resource within the guidelines of the HSRC.

Procedure for conflict resolution: All issues pertaining to the grant will be discussed and resolved within the Project Executive Committee. This process will, as with previous current collaboration, be designed to arrive at mutually agreeable solutions. The chairperson of the respective committee will make the final decision on issues raised within the committee that have not been resolved by discussion and simple vote.

**Resource and Data Sharing**

The potential for professional publications and presentations by all team members will be strongly encouraged. Joint presentations at professional meetings in the US, South Africa or internationally will be encouraged. Among the team, a collaborative writing taskforce for publication will be developed. The development of presentations and publications provide opportunities for individual team members to take the lead in dissemination (e.g., members who wish to serve as lead author). Publications benefit not only the disciplinary audiences and patient population, but also the team members.

Dissemination of findings will be made to the community participants and stakeholders. Recommendations for future interventions will be developed with input of stakeholders who provided initial information on PMTCT and HIV prevention related issues. The US team and the South Africa team will collaborate and encourage community stakeholders to participate in dissemination and development of related research. Utilizing the data from this preliminary study, the collaborators will compile a research review document for the Government of South Africa. Successful results of this research will be used to develop a translational proposal based on empirical results.

The study results will be disseminated through Dr. Peltzer, the South Africa PI. All investigators will work within the structure of the South Africa community sites to provide feedback to other medical providers and to the professional community by presentation of the results. Access to the database will be available under the supervision of the collaborators.

**References**

1. Abrahams N, Jewkes R. (2012). Managing and resisting stigma: a qualitative study among people living with HIV in South Africa. *J Int AIDS Soc*, 15, 17330.

2. Ackermann L, de Klerk K. (2002). Social factors that make South African women vulnerable to HIV infection. *Health Care Women Int*, 3, 163 - 72.

3. African Development Bank. (2009). The development of harmonized minimum standards for guidance on HIV testing and counseling and prevention of mother-to-child transmission of HIV in the SADC Region. PMTCT Country Report: ESOTHO. Available at: <http://www.hsrc.ac.za/research/output/outputDocuments/6312_Agu_PMTCT_Lesotho.pdf>.

4. Aluisio A, Richardson BA, Bosire R, John-Stewart G, Mbori-Ngacha D, Farquhar C. (2011). Male antenatal attendance and HIV testing are associated with decreased infant HIV infection and increased HIV-free survival. *Journal of Acquired Immune Deficiency Syndromes*, 56, 76-82.

5. Amico, K. (2006). Efficacy of antiretroviral therapy adherence interventions: A research synthesis of trials, 1996 to 2004. *Journal of Acquired Immune Deficiency Syndromes*, 41, 285-97.

6. Amico, K. (2011). A situated-Information motivation behavioral skills model of care initiation and maintenance (sIMB-CIM): An IMB model based approach to understanding and intervening in engagement in care for chronic medical conditions. *Journal of Health Psychology*, 16, 1071-1081.

7. Auvinen, Jaana, Tarja Suominen, and Maritta Valimaki. (2010). Male Participation and Prevention of Human Immunodeficiency Virus (HIV) Mother-to-child Transmission in Africa. *Psychology, Health & Medicine,* 15, 288-313.

8. Bajunirwe, F, Muzoora, M (2005). Barriers to the implementation of programs for the prevention of mother-to-child transmission of HIV: A cross-sectional survey in rural and urban Uganda. *AIDS Research and Therapy, 2*: 102, 1-7.

[9. Bancheno WM](http://www.ncbi.nlm.nih.gov/pubmed?term=Bancheno%20WM%5BAuthor%5D&cauthor=true&cauthor_uid=20824565), [Mwanyumba F](http://www.ncbi.nlm.nih.gov/pubmed?term=Mwanyumba%20F%5BAuthor%5D&cauthor=true&cauthor_uid=20824565), [Mareverwa J](http://www.ncbi.nlm.nih.gov/pubmed?term=Mareverwa%20J%5BAuthor%5D&cauthor=true&cauthor_uid=20824565). (2010). Outcomes and challenges of scaling up comprehensive PMTCT services in rural Swaziland, Southern Africa. [*AIDS Care.*](http://www.ncbi.nlm.nih.gov/pubmed/20824565)22:1130-5.

10. Barreiro P, Castilla JA, Labarga P, Soriano V. (2007). Is natural conception a valid option for HIV-serodiscordant couples? *Human Reproduction,* 22, 2353-2358*.*

11. Barron P, Pillay Y, Doherty T, Sherman G, Jackson D, Bhardwaj S, Robinson P, Goga A. (2013). Eliminating mother-to-child HIV transmission in South Africa. *Bull World Health Organ, 91*:70-4.

12. Becker, S., Mlay, R., Schwandt, H.M., Lyamuya, E. (2009). [Comparing Couples' and Individual Voluntary Counseling and Testing for HIV at Antenatal Clinics in Tanzania: A Randomized Trial.](http://www.ncbi.nlm.nih.gov/pubmed/19763813?itool=EntrezSystem2.PEntrez.Pubmed.Pubmed_ResultsPanel.Pubmed_RVDocSum&ordinalpos=5) *AIDS & Behavior*, 14, 558-66.

13. Becquet R, Dabis F. (2012). Turning the tide on HIV in women and children: Preventing breast-milk HIV transmission while increasing maternal life expectancy. *Clin Infect Dis*, 56, 140-142.

14. Bekker LG, Black V, Myer L, Rees H, Cooper D, Mall S, Mnyami C, Conradie F, Mahabeer I, Gilbert L, Schwartz S. (2011). Guideline on safer conception in fertile HIV-infected individuals and couples. *S Afr J HIV Med*, 12, 31-44.

15. Bouillon, K., Lert, F., Sitta, R., Schmaus, A., Spire, B., Dray-Spira, R. (2007). Factors correlated with disclosure of HIV infection in the French Antilles and French Guiana: results from the ANRS-EN13-VESPA-DFA study. *AIDS*, 21, 89 – 94.

16. Brusamento S, Ghanotakis E, Tudor Car L, van-Velthoven MH, Majeed A, Car J. (2012). Male involvement for increasing the effectiveness of prevention of mother-to-child HIV transmission (PMTCT) programmes. *Cochrane Database Syst Rev,* 10, CD009468.

17. Byamugisha R, Tumwine J, Semiyaga N, Tylleskar T. (2010). Determinants of male involvement in the prevention of mother-to-child transmission of HIV programme in Eastern Uganda: a cross-sectional survey. *Reproductive Health,* 7, 12.

18. Chetty T, Knight S, Giddy J, Crankshaw TL, Butler LM, Newell ML. (2012). A retrospective study of Human Immunodeficiency Virus transmission, mortality and loss to follow-up among infants in the first 18 months of life in a prevention of mother-to-child transmission programme in an urban hospital in KwaZulu-Natal, South Africa. *BMC Pediatr*, 12, 146.

19. Ciaranello AL, Perez F, Keatinge J, Park JE, Engelsmann B, Maruva M, Walensky RP, Dabis F, Chu J, Rusibamayila A, Mushavi A, Freedberg KA. (2012). What Will It Take to Eliminate Pediatric HIV? Reaching WHO Target Rates of Mother-to-Child HIV Transmission in Zimbabwe: A Model-Based Analysis. *PLoS Med*, 9, e1001156.

20. Clouse K, Pettifor A, Shearer K, Maskew M, Bassett J, Larson B, Van Rie A, Sanne I, Fox MP. (2013). Loss to follow-up before and after delivery among women testing HIV positive during pregnancy in Johannesburg, South Africa*. Trop Med Int Health, 18*: 451-460.

21. Cohen, M., Chen, Y., McCauley, M., Gamble, T., et al. (2011). Prevention of HIV-1 infection with early antiretroviral therapy. *New England Journal of Medicine*, 365, 493-505.

22. Colvin M, Chopra M, Doherty T, Jackson D, Levin J, Willumsen J, Goga A, Moodley P for the Good Start Study Group. (2007). Operational effectiveness of single-dose nevirapine in preventing mother-to-child transmission of HIV. *Bulletin of the World Health Organization |* *85,* 466-473.

[23. Conkling M](http://www.ncbi.nlm.nih.gov/pubmed?term=%22Conkling%20M%22%5BAuthor%5D), [Shutes EL](http://www.ncbi.nlm.nih.gov/pubmed?term=%22Shutes%20EL%22%5BAuthor%5D), [Karita E](http://www.ncbi.nlm.nih.gov/pubmed?term=%22Karita%20E%22%5BAuthor%5D), [Chomba E](http://www.ncbi.nlm.nih.gov/pubmed?term=%22Chomba%20E%22%5BAuthor%5D), [Tichacek A](http://www.ncbi.nlm.nih.gov/pubmed?term=%22Tichacek%20A%22%5BAuthor%5D), [Sinkala M](http://www.ncbi.nlm.nih.gov/pubmed?term=%22Sinkala%20M%22%5BAuthor%5D), [Vwalika B](http://www.ncbi.nlm.nih.gov/pubmed?term=%22Vwalika%20B%22%5BAuthor%5D), [Iwanowski M](http://www.ncbi.nlm.nih.gov/pubmed?term=%22Iwanowski%20M%22%5BAuthor%5D), [Allen SA](http://www.ncbi.nlm.nih.gov/pubmed?term=%22Allen%20SA%22%5BAuthor%5D). (2010). Couples' voluntary counselling and testing and nevirapine use in antenatal clinics in two African capitals: a prospective cohort study. *Journal of the International AIDS Society*, 13, 10.

[24. Cooke GS](http://www.ncbi.nlm.nih.gov/pubmed?term=Cooke%20GS%5BAuthor%5D&cauthor=true&cauthor_uid=19771168), [Little KE](http://www.ncbi.nlm.nih.gov/pubmed?term=Little%20KE%5BAuthor%5D&cauthor=true&cauthor_uid=19771168), [Bland RM](http://www.ncbi.nlm.nih.gov/pubmed?term=Bland%20RM%5BAuthor%5D&cauthor=true&cauthor_uid=19771168), [Thulare H](http://www.ncbi.nlm.nih.gov/pubmed?term=Thulare%20H%5BAuthor%5D&cauthor=true&cauthor_uid=19771168), [Newell ML](http://www.ncbi.nlm.nih.gov/pubmed?term=Newell%20ML%5BAuthor%5D&cauthor=true&cauthor_uid=19771168). (2009). Need for timely paediatric HIV treatment within primary health care in rural South Africa. [*PLoS One.*](http://www.ncbi.nlm.nih.gov/pubmed/19771168) 2009 Sep 22;4(9):e7101. doi: 0.1371/journal.pone.0007101.

25. Dageid W, Govender K, Gordon SF. (2012). Masculinity and HIV disclosure among heterosexual South African men: implications for HIV/AIDS intervention. *Cult Health Sex*, 14, 925-940.

26. Department of Health, Pretoria. (2007). *National Antenatal Sentinel HIV and Syphilis Prevalence Survey in South Africa, 2007*. Pretoria, South Africa: Department of Health.

27. Department of Health, Pretoria. (2010). *National Antenatal Sentinel HIV and Syphilis Prevalence Survey in South Africa, 2009*. Pretoria, South Africa: Department of Health.

28. Department of Health, Pretoria. (2011). *National Antenatal Sentinel HIV and Syphilis Prevalence Survey in South Africa, 2010*. Pretoria, South Africa: Department of Health.

29. Deribe K, Woldemichael K, Njau BJ, Yakob B, Biadgilign S, Amberbir A. (2010). Gender differences regarding barriers and motivators of HIV status disclosure among HIV-positive service users. *SAHARA Journal,* 7, 30-39.

30. Deribe K, Woldemichael K, Wondafrash M, Haile A, Amberbir A. (2008). Disclosure experience and associated factors among HIV positive men and women clinical service users in southwest Ethiopia. *BMC Public Health,* 8, 81.

[31. Desclaux A](http://www.ncbi.nlm.nih.gov/pubmed?term=Desclaux%20A%5BAuthor%5D&cauthor=true&cauthor_uid=19559512), [Alfieri C](http://www.ncbi.nlm.nih.gov/pubmed?term=Alfieri%20C%5BAuthor%5D&cauthor=true&cauthor_uid=19559512). (2009). Counseling and choosing between infant-feeding options: overall limits and local interpretations by health care providers and women living with HIV in resource-poor countries (Burkina Faso, Cambodia, Cameroon). [*Social Science and Medicine,*](http://www.ncbi.nlm.nih.gov/pubmed/19559512) 69(6), 821-9.

32. Desgrées-du-Loû, A., Brou, H., Traore, A.T., Djohan, G., Becquet, R., & Leroy V. (2009). [From prenatal HIV testing of the mother to prevention of sexual HIV transmission within the couple.](http://www.ncbi.nlm.nih.gov/pubmed/19552991?itool=EntrezSystem2.PEntrez.Pubmed.Pubmed_ResultsPanel.Pubmed_RVDocSum&ordinalpos=8) *Social Science & Medicine*, 69, 892-9.

33. Dommering CJ, Garvelink MM, Moll AC, van Dijk J, Imhof SM, Meijers-Heijboer H, Henneman L. (2012). Reproductive behavior of individuals with increased risk of having a child with retinoblastoma. *Clinical Genetics,* 81, 216–223.

34. Dube Q, Dow A, Chirambo C, Lebov J, Tenthani L, Moore M, Heyderman RS, Van Rie A; for the CHIDEV study team. (2012). Implementing early infant diagnosis of HIV infection at the primary care level: experiences and challenges in Malawi. *Bull World Health Organ,* 90, 699-704.

35. Dunkle KL, Jewkes RK, Brown HC, Gray GE, McIntryre JA, Harlow SD. (2004). Gender-based violence, relationship power, and risk of HIV infection in women attending antenatal clinics in South Africa. *Lancet*, 363, 1415-1421.

36. Ekstrand M, Chandy S, Heylen E, Steward W, Singh G. (2010) Developing useful HAART adherence measures for India: The Prerana Study. *Journal of Acquired Immune Deficiency Syndromes,* 53, 415-416.

37. EngenderHealth and UNFPA. (2008). *Sexual and Reproductive Health of Women and Adolescent Girls Living with HIV: Guidance for Health Managers, Health Workers, and Activists*. Nova York: EngenderHealth and Brasília, DF: UNFPA.

38. Expert Committee (2011) Prevention of Mother-to-Child Transmission of HIV: Expert Panel Report and Recommendations to the U.S. Congress and U.S. Global AIDS Coordinator, January 2010. Retrieved at www.pepfar.gov/documents/organization/135465.pdf

39. Falnes EF, Moland KM, Tyllerskar T, de Paoli MM, Msuya SE, Engebretsen IMS. (2011). “It is her responsibility”: partner involvement in prevention of mother to child transmission of HIV programmes, northern Tanzania. *Journal of the International AIDS Society*, 14, 21–32.

40. Farquhar C, James K, Barbra R, Marjory K, Francis J, Ruth N, Dorothy M, Grace S. (2004). Antenatal couple counseling increases uptake of interventions to prevent HIV-1 transmission. *Journal of Acquired Immune Deficiency Syndromes*, 37, 1620-6.

41. Fisher J, Amico R, Fisher W, Harman J. (2008). The information-motivation-behavioral skills model of antiretroviral adherence and its implications. *Current HIV/AIDS Reports*, 5, 193-203.

42. Fisher J, Fisher W, Amico K, Harman J. (2006). An information-motivation-behavioral skills model of adherence to antiretroviral therapy. *Health Psychology*, 25, 462-473.

43. Fisher JD, Fisher WA, Cornman DH, Amico KR, Bryan A, Friedland GH. (2006). Clinician-delivere intervention during routine clinical care reduces unprotected sexual behavior among HIV-infected patients. *Journal of Acquired Immune Deficiency Syndromes*, 41, 44-52.

44. Fisher JD, Fisher WA, Misovich SJ, Kimble DL, Malloy TE. (1996). Changing AIDS risk behavior: Effects of an intervention emphasizing AIDS risk reduction information, motivation, and behavioral skills in a college student population. *Health Psychology*, 15, 114–123.

45. Ghanotakis E, Peacock D, Wilcher R. (2012). The importance of addressing gender inequality in efforts to end vertical transmission of HIV. *J Int AIDS Soc*, 15 suppl 2, 17385

46. Glasgow, E. R. (2003). Translating research to practice. *Diabetes Care*, 26, 2451-2456.

47. Goga A, Dinh TH, Dlamini N, Mosala T, Lombard C, Puren A, Sherman G, Crowley S, Woldesenbet S, Solomon W, Kula N, Ramokolo V, Pillay Y, Jackson D, South Africa PMTCT Effectiveness Survey (SAPMTCTE) team. (2011). Impact of the national prevention of mother to child transmission (PMTCT) program on mother-to-child transmission of HIV (MTCT), South Africa, 2010 (abstract no. MOAC0206). *6th International AIDS Society Conference on HIV Pathogenesis, treatment and prevention*. Rome, Italy.

48. Goga AE, Dinh TH, Jackson DJ for the SAPMTCTE Study Group. (2012). Evaluation of the Effectiveness of the national Prevention of Mother-to-Child Transmission (PMTCT) Programme on Infant HIV Measured at Six Weeks Postpartum in South Africa, 2010. South African Medical Research Council, National Department of Health of South Africa and PEPFAR/US Center for Disease Control and Prevention. Available at: <http://doh.gov.za/docs/reports/2012/pmtcteffectiveness.pdf>

49. Goga AE, Doherty T, Jackson DJ, Sanders D, Colvin M, Chopra M, Kuhn L. (2012). Infant feeding practices at routine PMTCT sites, South Africa: results of a prospective observational study amongst HIV exposed and unexposed infants - birth to 9 months. *Int Breastfeed J*, 7, 4.

50. Grimwood A, Fatti G, Mothibi E, Eley B, Jackson D. (2012). Progress of preventing mother-to-child transmission of HIV at primary healthcare facilities and district hospitals in three South African provinces. *South African Medical Journal*, 102, 81-83.

51. Groves AK, Kagee A, Maman S, Moodley D, Rouse P. (2011). Associations Between Intimate Partner Violence and Emotional Distress Among Pregnant Women in Durban, South Africa. *Journal of Interpersonal Violence*, Dec 26. [Epub ahead of print].

52. Hardon A, Vernooij E, Bongololo-Mbera G, Cherutich P, Desclaux A, Kyaddondo D, Ky-Zerbo O, Neuman M, Wanyenze R, Obermeyer C. (2012). Women's views on consent, counseling and confidentiality in PMTCT: a mixed-methods study in four African countries. *BMC Public Health*, Jan 11. [Epub ahead of print].

53. Harrington EK, Newmann SJ, Onono M, Schwartz KD, Bukusi EA, Cohen CR, Grossman D. (2012). Fertility Intentions and Interest in Integrated Family Planning Services among Women Living with HIV in Nyanza Province, Kenya: A Qualitative Study. *Infect Dis Obstet Gynecol*, 2012, 809682.

54. Health Systems Trust. (2009). The district health barometer – year 2007/08 (Technical Report). Durban, South Africa. Available at: http://www.hst.org.za/publications/850

55. Holt K, Lince N, Hargey A, Struthers H, Nkala B, Mclntyre J, Gray G, Mnyani C, Blanchard K. (2012). Assessment of service availability and health care workers' opinions about young women's sexual and reproductive health in Soweto, South Africa. *Afr J Reprod Health*, 16, 283-93.

56. Holzemer W, Uys L, Chirwa M, Greeff M, Makoae L, Kohi T, Dlamini P, Stewart A, Mullan J, Phetlhu R, Wantland D, Durrheim K. (2007). Validation of the HIV/AIDS stigma instrument. *AIDS Care,* 19, 1002-1012.

[57. Horwood C](http://www.ncbi.nlm.nih.gov/pubmed?term=Horwood%20C%5BAuthor%5D&cauthor=true&cauthor_uid=20561313), [Haskins L](http://www.ncbi.nlm.nih.gov/pubmed?term=Haskins%20L%5BAuthor%5D&cauthor=true&cauthor_uid=20561313), [Vermaak K](http://www.ncbi.nlm.nih.gov/pubmed?term=Vermaak%20K%5BAuthor%5D&cauthor=true&cauthor_uid=20561313), [Phakathi S](http://www.ncbi.nlm.nih.gov/pubmed?term=Phakathi%20S%5BAuthor%5D&cauthor=true&cauthor_uid=20561313), [Subbaye R](http://www.ncbi.nlm.nih.gov/pubmed?term=Subbaye%20R%5BAuthor%5D&cauthor=true&cauthor_uid=20561313), [Doherty T](http://www.ncbi.nlm.nih.gov/pubmed?term=Doherty%20T%5BAuthor%5D&cauthor=true&cauthor_uid=20561313). (2010). Prevention of mother to child transmission of HIV (PMTCT) programme in KwaZulu-Natal, South Africa: an evaluation of PMTCT implementation and integration into routine maternal, child and women's health services. *Trop Med Int Health*, 15:992-9.

58. Hudgens MG, Taha TE, Omer SB, Jamieson DJ, Lee H, Mofenson LM, Chasela C, Kourtis AP, Kumwenda N, Ruff A, Bedri A, Jackson JB, Musoke P, Bollinger RC, Gupte N, Thigpen MC, Taylor A, van der Horst C. (2012). Pooled Individual Data Analysis of Five Randomized Trials of Infant Nevirapine Prophylaxis to Prevent Breast-Milk HIV-1 Transmission. *Clin Infect Dis*, 56, 131-139.

59. Hyginus E, Chukwuemeka I, Lawrence I, Sunday M. (2012). HIV-related intimate partner violence among pregnant women in Nigeria. *East Afr J Public Health*, 9, 29-32.

60. Idonije, BO, Oluba, OM & Otamere, HO. (2011). A study on knowledge, attitude and practice of contraception among secondary school students. *JPCS,* 2, 22-34.

61. Jones, D. (1998). Conceptual structure of HIV+ women with PTSD: Trauma construct elaboration. Dissertation. University of North Texas.

62. Jones, DL, Peltzer, K, Villar-Loubet, O, Shikwane, E, Cook, R, Vamos, S, Weiss, SM (2013). Reducing the risk of HIV infection during pregnancy among South African women. AIDS Care. PMID 23438041

63. Jones, D., Ross, D., Weiss, S.M., Bhat, G., & Chitalu, N. (2005). Influence of partner participation on sexual risk behavior reduction among HIV-positive Zambian women. *Journal of Urban Health*, 82, 92-100.

64. Jones, D., Villar-Loubet, O., Kankasa, C., Chitalu, N., Mumbi, M., & Weiss, S. (2010). Contraception and family planning among HIV seroconcordant and serodiscordant couples in the US and Zambia. *Open Access Journal of Contraception*.

65. Jones, D., Weiss, S.M., Bhat, G.J., Feldman, S.A., Bwalya, V., & Budash, D. (2004). A Sexual Barrier Intervention for HIV+/- Zambian Women: Acceptability and Use of Vaginal Chemical Barriers. *Journal of Multicultural Nursing and Health*, 10, 27-31.

66. Jones, D., Weiss, S.M., Malow, R.M., Ishii, M., Devieux, J., Stanley, H., Cassells, A., Tobin, J.N., Brondolo, E., LaPerriere, A., Efantis-Potter, J., O’Sullivan, M.J., & Schneiderman, N. (2001). A Brief Sexual Barrier Intervention for Women Living with AIDS: Acceptability, Use and Ethnicity. *Journal of Urban Health*, 78, 593-604.

67. Jones, D.L., Bhat, G.J., Weiss, S.M., Feldman, D.A., & Bwalya, V. (2006). Influencing Sexual Practices among HIV Positive Zambian Women. *AIDS Care*, 18, 629-635.

68. Jones, D.L., Weiss, S.M., Chitalu, N., Bwalya, V., & Villar, O. (2008a). Acceptability of Microbicidal Surrogates among Zambian Women. *Sexually Transmitted Diseases*, 35, 147-153.

69. Jones, D.L., Weiss, S.M., Chitalu, N., Kumar, M., Villar, O., Bwalya, V., & Mumbi, M. (2007). Sexual risk intervention in multiethnic drug and alcohol users. *American Journal of Infectious Diseases*, 3, 169-176.

70. Jones, D.L., Weiss, S.M., Chitalu, N., Mumbi, M., Shine, A., Vamos, S., Villar, O. (2008b). Acceptability and Use of Sexual Barrier Products and Lubricants among HIV Seropositive Zambian Men. *AIDS Patient Care & STDs*, 22, 1015-1020.

71. Jones, D.L., Weiss, S.M., Chitalu, N., Mumbi, M., Vamos, S., Villar-Loubet, O., & Waldrop-Valverde, D. (2009). Sexual Risk Reduction among Zambian Couples. *SAHARA*, 6, 69-75.

72. Jones, D.L., Weiss, S.M., Waldrop-Valverde, D., Chitalu, N., Mumbi, M., & Vamos, S. (2008c). Community-Based Risk Reduction in Zambia. *Open Health Services and Policy Journal*, 1, 38-44.

73. Jones DL, Zulu I, Vamos S, Cook R, Chitalu N, Weiss SM. (2012). Determinants of Engagement in HIV Treatment and Care Among Zambians New to Antiretroviral Therapy. *J Assoc Nurses AIDS Care*. [Epub ahead of print]

[74. Jones](http://www.tandfonline.com/action/doSearch?action=runSearch&type=advanced&result=true&prevSearch=%2Bauthorsfield%3A(Jones%2C+S.+A.)) SA, [Sherman](http://www.tandfonline.com/action/doSearch?action=runSearch&type=advanced&result=true&prevSearch=%2Bauthorsfield%3A(Sherman%2C+G.+G.)), GG, [Varga](http://www.tandfonline.com/action/doSearch?action=runSearch&type=advanced&result=true&prevSearch=%2Bauthorsfield%3A(Varga%2C+C.+A.)), CA. (2005). Exploring socio-economic conditions and poor follow-up rates of HIV-exposed infants in Johannesburg, South Africa. *AIDS Care, 17*: 466-470.

75. Joseph, D., Project San Francisco, Rwanda-Zambia HIV Research Group, & Emory University Rollins School of Public Health, Kigali, Rwanda. (2004). Improving on a Successful Model for Promoting Couples’ VCT in Two African Capitals: Mobile Couples’ HIV Testing Units. *Proceedings of XV International AIDS Conference*, Bangkok, Thailand.

76. Kalichman, S.C., Rompa, D., & Cage M. (2005). Group intervention to reduce HIV transmission risk behavior among persons. *Behavior Modification*, 29, 256-85.

77. Katz DA, Kiarie JN, John-Stewart GC, Richardson BA, John FN, Farquhar C. (2009b). [HIV testing men in the antenatal setting: Understanding male non-disclosure.](http://www.ncbi.nlm.nih.gov/pubmed/19833691?itool=EntrezSystem2.PEntrez.Pubmed.Pubmed_ResultsPanel.Pubmed_RVDocSum&ordinalpos=4) *International Journal of STD & AIDS*, 20, 765-7.

78. Katz, D.A., Kiarie, J.N., John-Stewart, G.C., Richardson, B.A., John, F.N., & Farquhar, C. (2009a). [Male perspectives on incorporating men into antenatal HIV counseling and testing.](http://www.ncbi.nlm.nih.gov/pubmed/19881884?itool=EntrezSystem2.PEntrez.Pubmed.Pubmed_ResultsPanel.Pubmed_RVDocSum&ordinalpos=3) *PLoS One*, 4, e7602.

79. Kieffer, M.P., et al. (2010). Repeat HIV testing in labor and delivery as a standard of care increases ARV provision for women who seroconvert during pregnancy (abstract no. 156). *Proceedings of the 17th Annual Conference on Retroviruses and Opportunistic Infections (CROI).* San Francisco, C.A.

80. Kim HY, Kasonde P, Mwiya M, Thea DM, Kankasa C, Sinkala M, Aldrovandi G, Kuhn L. (2012). Pregnancy loss and role of infant HIV status on perinatal mortality among HIV-infected women. *BMC Pediatr*, 12, 138.

81. Kim MH, Ahmed S, Buck WC, Preidis GA, Hosseinipour MC, Bhalakia A,Nanthuru D, Kazembe PN, Chimbwandira F, Giordano TP, Chiao EY,Schutze GE, Kline MW. (2012). The Tingathe programme: a pilot intervention using community health workers to create a continuum of care in the prevention of mother to child transmission of HIV (PMTCT) cascade of services in Malawi. *J Int AIDS Soc*, 15(4), 1-11.

[82. Klar N](http://www.ncbi.nlm.nih.gov/pubmed?term=%22Klar%20N%22%5BAuthor%5D), [Donner A](http://www.ncbi.nlm.nih.gov/pubmed?term=%22Donner%20A%22%5BAuthor%5D). (2001) Current and future challenges in the design and analysis of cluster randomization trials. *Statistics in Medicine*, 20, 3729-3740.

83. Kromdijk W, Mulder JW, Rosing H, Smit PM, Beijnen JH, Huitema AD. (2012). Use of dried blood spots for the determination of plasma concentrations of nevirapine and efavirenz. *Journal of Antimicrobial Chemotherapy*, 67, 1211-1216.

84. Kumar SB, Rice CE, Milner DA Jr, Ramirez NC, Iv WE, Mwapasa V, Turner AN, Kwiek JJ. (2012). Elevated cytokine and chemokine levels in the placenta are associated with in utero HIV-1 mother-to-child transmission. *AIDS*, 26, 685-694.

85. Kuonza LR, Tshuma CD, Shambira GN, Tshimanga M. (2011). Non-adherence to the single dose nevirapine regimen for the prevention of mother-to-child transmission of HIV in Bindura town, Zimbabwe: a cross-sectional analytic study. *BMC Public Health*, 10, 218.

[86. Ladzani R](http://www.ncbi.nlm.nih.gov/pubmed?term=%22Ladzani%20R%22%5BAuthor%5D), [Peltzer K](http://www.ncbi.nlm.nih.gov/pubmed?term=%22Peltzer%20K%22%5BAuthor%5D), [Mlambo MG](http://www.ncbi.nlm.nih.gov/pubmed?term=%22Mlambo%20MG%22%5BAuthor%5D), [Phaweni K](http://www.ncbi.nlm.nih.gov/pubmed?term=%22Phaweni%20K%22%5BAuthor%5D). (2011). Infant-feeding practices and associated factors of HIV-positive mothers at Gert Sibande, South Africa. *Acta Paediatrica,* 100, 538-542.

87. Laher F, Cescon A, Lazarus E, Kaida A, Makongoza M, Hogg RS, Soon CN, Miller CL, Gray G. (2012). Conversations With Mothers: Exploring Reasons for Prevention of Mother-to-Child Transmission (PMTCT) Failures in the Era of Programmatic Scale-Up in Soweto, South Africa. *AIDS & Behavior,* 16, 91-98.

88. Landes M, van Lettow M, Bedell R, Mayuni I, Chan AK, Tenthani L, Schouten E. (2012). Mortality and Health Outcomes in HIV-Infected and HIV-Uninfected Mothers at 18-20 Months Postpartum in Zomba District, Malawi*. PLoS One*, 7, e44396.

89. Langen TT. (2005). Gender power imbalance on women’s capacity to negotiate self-protection against HIV/AIDS in Botswana and South Africa. *African Health Sciences*, 5, 188–197.

90. Larsson EC, Thorson AE, Pariyo G, Waiswa P, Kadobera D, Marrone G, Ekström AM. (2012). Missed Opportunities: Barriers to HIV Testing during Pregnancy from a Population Based Cohort Study in Rural Uganda. *PLoS One,* 7(8), e37590.

91. Lazarus R, Struthers H, Violari A. (2013). Promoting safe infant feeding practices - the importance of structural, social and contextual factors in Southern Africa. *J Int AIDS Soc, 16*:18037.

92. Letshwenyo-Maruatona, S. (2012). Male participation in sexual and reproductive health. Gaberone, Botswana: PhD Thesis, Department of Educational Foundations, University of Botswana.

93. Lindegren ML, Kennedy CE, Bain-Brickley D, Azman H, Creanga AA, Butler LM, Spaulding AB, Horvath T, Kennedy GE. (2012). Integration of HIV/AIDS services with maternal, neonatal and child health, nutrition, and family planning services. *Cochrane Database Syst Rev*, 9, CD010119.

94. Little KM, Kilmarx PH, Taylor AW, Rose CE, Rivadeneira ED, Nesheim SR. (2012). A Review of Evidence for Transmission of Human Immunodeficiency Virus from Children to Breastfeeding Women and Implications for Prevention. *Pediatr Infect Dis J*, 31, 938-942.

95. Lopez LM, Hiller JE, Grimes DA. (2010). Education for contraceptive use by women after childbirth. *Cochrane Database Syst Rev,* 20, CD001863.

96. Loutfy M, Raboud J, Wong J, Yudin M, Diong C, Blitz S, Margolese S, Hart T, Ogilvie G, Masinde K, Tharao W, Linklater G, Salam K, Ongoiba F, Angel J, Smaill F, Rachlis A, Ralph E, Walmsley S. (2012). High prevalence of unintended pregnancies in HIV- positive women of reproductive age in Ontario, Canada: a retrospective study. *HIV Medicine*, 13, 107-117.

97. Makanani (2009) [Makayoto LA](http://www.ncbi.nlm.nih.gov/pubmed?term=Makayoto%20LA%5BAuthor%5D&cauthor=true&cauthor_uid=22569943), [Omolo J](http://www.ncbi.nlm.nih.gov/pubmed?term=Omolo%20J%5BAuthor%5D&cauthor=true&cauthor_uid=22569943), [Kamweya AM](http://www.ncbi.nlm.nih.gov/pubmed?term=Kamweya%20AM%5BAuthor%5D&cauthor=true&cauthor_uid=22569943), [Harder VS](http://www.ncbi.nlm.nih.gov/pubmed?term=Harder%20VS%5BAuthor%5D&cauthor=true&cauthor_uid=22569943), [Mutai J](http://www.ncbi.nlm.nih.gov/pubmed?term=Mutai%20J%5BAuthor%5D&cauthor=true&cauthor_uid=22569943). (2012). Prevalence and Associated Factors of Intimate Partner Violence Among Pregnant Women Attending Kisumu District Hospital, Kenya. [*Matern Child Health J.*](http://www.ncbi.nlm.nih.gov/pubmed/22569943) *2012 May 9.* [Epub ahead of print]

98. Maman S, Cathcart R, Burkhardt G, Omba S, Thompson D. (2012). The infant feeding choices and experiences of women living with HIV in Kinshasa, Democratic Republic of Congo. *AIDS Care*, 24, 259-265.

99. Matthews LT, Crankshaw T, Giddy J, Kaida A, Psaros C, Ware NC, Smit JA, Bangsberg DR. (2012). Reproductive Counseling by Clinic Healthcare Workers in Durban, South Africa: Perspectives from HIV-Infected Men and Women Reporting Serodiscordant Partners*. Infect Dis Obstet Gynecol*, 2012, 146348.

100. Matthews LT, Mukherjee JS. (2009) Strategies for harm reduction among HIV-affected couples who want to conceive. *AIDS & Behavior,* 13, 5-11*.*

101. Matthews LT, Smit JA, Cu-Uvin S, Cohan D. (2012). Antiretrovirals and safer conception for HIV-serodiscordant couples. *Curr Opin HIV AIDS*, 7, 569-578.

102. Melendez, R.M., Hoffman, S., Exner, T., Leu, C., & Ehrhardt, A.A. (2003). Intimate Partner Violence and Safer sex negotiation: effects of a Gender-Specific Intervention. *Archives of Sexual Behaviour*, 32, 499 – 511.

103. Mepham, S., Z. Zondi, A. Mbuyazi, N. Mkhwanazi and M. L. Newell. (2011). Challenges in PMTCT antiretroviral adherence in northern KwaZulu-Natal, South Africa. *AIDS Care*, 23, 741-747.

104. Meyer-Bahlburg, H.F.L., Ehrhardt, A. A., Exner, T. M., Gruen, R. S., & Dugan, T. (1995). The Sexual Risk Behavior Schedule for Depressed Youths, Female, Baseline (SERBAS-Y-DEPRF-1). Unpublished manuscript, NYS Psychiatric Institute & Department of Psychiatry, Columbia University.

105. Mmeje O, Cohen CR, Cohan D. (2012). Evaluating Safer Conception Options for HIV-Serodiscordant Couples (HIV-Infected Female/HIV- Uninfected Male): A Closer Look at Vaginal Insemination. *Infect Dis Obstet Gynecol*, 2012, 587651.

106. Mohlala BK, Boily MC, Gregson S. (2011). The forgotten half of the equation: randomized controlled trial of a male invitation to attend couple voluntary counselling and testing. *AIDS*, 25, 1535-1541.

107. Montgomery E, van der Straten A, Torjesen K. (2011). "Male involvement" in women and children's HIV prevention: challenges in definition and interpretation. *J Acquir Immune Defic Syndr, 5*7(5), e114-116; author reply e116-117.

108. Moses S, Tomlinson M. (2012). The fluidity of disclosure: A longitudinal exploration of women's experience and understanding of HIV disclosure in the context of pregnancy and early motherhood. *AIDS Care*. [Epub ahead of print]

[109. Mugo NR](http://www.ncbi.nlm.nih.gov/pubmed?term=%22Mugo%20NR%22%5BAuthor%5D), [Heffron R](http://www.ncbi.nlm.nih.gov/pubmed?term=%22Heffron%20R%22%5BAuthor%5D), [Donnell D](http://www.ncbi.nlm.nih.gov/pubmed?term=%22Donnell%20D%22%5BAuthor%5D), [Wald A](http://www.ncbi.nlm.nih.gov/pubmed?term=%22Wald%20A%22%5BAuthor%5D), [Were EO](http://www.ncbi.nlm.nih.gov/pubmed?term=%22Were%20EO%22%5BAuthor%5D), [Rees H](http://www.ncbi.nlm.nih.gov/pubmed?term=%22Rees%20H%22%5BAuthor%5D), [Celum C](http://www.ncbi.nlm.nih.gov/pubmed?term=%22Celum%20C%22%5BAuthor%5D), [Kiarie JN](http://www.ncbi.nlm.nih.gov/pubmed?term=%22Kiarie%20JN%22%5BAuthor%5D), [Cohen CR](http://www.ncbi.nlm.nih.gov/pubmed?term=%22Cohen%20CR%22%5BAuthor%5D), [Kayintekore K](http://www.ncbi.nlm.nih.gov/pubmed?term=%22Kayintekore%20K%22%5BAuthor%5D), [Baeten JM](http://www.ncbi.nlm.nih.gov/pubmed?term=%22Baeten%20JM%22%5BAuthor%5D); [Partners in Prevention HSV/HIV Transmission Study Team](http://www.ncbi.nlm.nih.gov/pubmed?term=%22Partners%20in%20Prevention%20HSV%2FHIV%20Transmission%20Study%20Team%22%5BCorporate%20Author%5D). (2011). Increased risk of HIV-1 transmission in pregnancy: a prospective study among African HIV-1-serodiscordant couples. *AIDS,* 25, 1887-1895.

110. Myer L, Zulliger R, Bekker LG, Abrams E. (2012). Systemic delays in the initiation of antiretroviral therapy during pregnancy do not improve outcomes of HIV-positive mothers: a cohort study. *BMC Pregnancy Childbirth*, 12, 94.

111. Nachega JB, Morroni C, Zuniga JM, Sherer R, Beyrer C, Solomon S, Schechter M, Rockstroh J. (2012). HIV-Related Stigma, Isolation, Discrimination, and Serostatus Disclosure: A Global Survey of 2035 HIV-Infected Adults. *J Int Assoc Physicians AIDS Care (Chic),* 11, 172-178.

112. Nassali, M, Nakanjako, D, Kyabayinze, D, Beyezaa, Okotha A, Mutyabaa, T. (2009). Access to HIV/AIDS care for mothers and children in sub-Saharan Africa: adherence to the postnatal PMTCT program. *AIDS Care*, 21, 1124-1131.

113. Natchu UC, Liu E, Duggan C, Msamanga G, Peterson K, Aboud S, Spiegelman D, Fawzi WW. (2012). Exclusive breastfeeding reduces risk of mortality in infants up to 6 mo of age born to HIV-positive Tanzanian women. *Am J Clin Nutr,* 96, 1071-1078.

114. National Department of Health (NDOH). (2012). The National Antenatal Sentinel HIV and Syphilis Prevalence Survey, South Africa, 2011. Pretoria: National Department of Health. Available at: http://www.doh.gov.za/docs/reports/2012/Antenatal_Sentinel_survey_Report2012_final.pdf.

115. National Health Laboratory Service. (2011). Mpumalanga Facility HIV PCR Report Dec 2011.

[116. Ndou T](http://www.ncbi.nlm.nih.gov/pubmed?term=Ndou%20T%5BAuthor%5D&cauthor=true&cauthor_uid=23364086), [van Zyl G](http://www.ncbi.nlm.nih.gov/pubmed?term=van%20Zyl%20G%5BAuthor%5D&cauthor=true&cauthor_uid=23364086), [Hlahane S](http://www.ncbi.nlm.nih.gov/pubmed?term=Hlahane%20S%5BAuthor%5D&cauthor=true&cauthor_uid=23364086), [Goudge J](http://www.ncbi.nlm.nih.gov/pubmed?term=Goudge%20J%5BAuthor%5D&cauthor=true&cauthor_uid=23364086). (2013). A rapid assessment of a community health worker pilot programme to improve the management of hypertension and diabetes in Emfuleni sub-district of Gauteng Province, South Africa. [*Glob Health Action,*](http://www.ncbi.nlm.nih.gov/pubmed/23364086) *6*:19228. doi: 10.3402/gha.v6i0.19228.

117. Nkuoh GN, Meyer DJ, Nshom EM. (2013). Women's Attitudes Toward Their Partners' Involvement in Antenatal Care and Prevention of Mother-to-Child Transmission of HIV in Cameroon, Africa. *J Midwifery Womens Health, 58*: 83-91.

[118. Nxumalo N](http://www.ncbi.nlm.nih.gov/pubmed?term=Nxumalo%20N%5BAuthor%5D&cauthor=true&cauthor_uid=23364101), [Goudge J](http://www.ncbi.nlm.nih.gov/pubmed?term=Goudge%20J%5BAuthor%5D&cauthor=true&cauthor_uid=23364101), [Thomas L](http://www.ncbi.nlm.nih.gov/pubmed?term=Thomas%20L%5BAuthor%5D&cauthor=true&cauthor_uid=23364101). (2013). Outreach services to improve access to health care in South Africa: lessons from three community health worker programmes. [*Glob Health Action,*](http://www.ncbi.nlm.nih.gov/pubmed/23364101) *6*:19283. doi: 10.3402/gha.v6i0.19283.

119. Ochola SA, Labadarios D, Nduati RW. (2012). Impact of counselling on exclusive breast-feeding practices in a poor urban setting in Kenya: a randomized controlled trial. *Public Health Nutr*, 8, 1-9.

[120. Orne-Gliemann J](http://www.ncbi.nlm.nih.gov/pubmed?term=%22Orne-Gliemann%20J%22%5BAuthor%5D), [Tchendjou PT](http://www.ncbi.nlm.nih.gov/pubmed?term=%22Tchendjou%20PT%22%5BAuthor%5D), [Miric M](http://www.ncbi.nlm.nih.gov/pubmed?term=%22Miric%20M%22%5BAuthor%5D), [Gadgil M](http://www.ncbi.nlm.nih.gov/pubmed?term=%22Gadgil%20M%22%5BAuthor%5D), [Butsashvili M](http://www.ncbi.nlm.nih.gov/pubmed?term=%22Butsashvili%20M%22%5BAuthor%5D), [Eboko F](http://www.ncbi.nlm.nih.gov/pubmed?term=%22Eboko%20F%22%5BAuthor%5D), [Perez-Then E](http://www.ncbi.nlm.nih.gov/pubmed?term=%22Perez-Then%20E%22%5BAuthor%5D), [Darak S](http://www.ncbi.nlm.nih.gov/pubmed?term=%22Darak%20S%22%5BAuthor%5D), [Kulkarni S](http://www.ncbi.nlm.nih.gov/pubmed?term=%22Kulkarni%20S%22%5BAuthor%5D), [Kamkamidze G](http://www.ncbi.nlm.nih.gov/pubmed?term=%22Kamkamidze%20G%22%5BAuthor%5D), [Balestre E](http://www.ncbi.nlm.nih.gov/pubmed?term=%22Balestre%20E%22%5BAuthor%5D), [du Loû AD](http://www.ncbi.nlm.nih.gov/pubmed?term=%22du%20Lo%C3%BB%20AD%22%5BAuthor%5D), [Dabis F](http://www.ncbi.nlm.nih.gov/pubmed?term=%22Dabis%20F%22%5BAuthor%5D). (2010). Couple-oriented prenatal HIV counseling for HIV primary prevention: an acceptability study. *BMC Public Health,* 10, 197.

[121. Peltzer K](http://www.ncbi.nlm.nih.gov/pubmed?term=%22Peltzer%20K%22%5BAuthor%5D), [Sikwane E](http://www.ncbi.nlm.nih.gov/pubmed?term=%22Sikwane%20E%22%5BAuthor%5D), [Majaja M](http://www.ncbi.nlm.nih.gov/pubmed?term=%22Majaja%20M%22%5BAuthor%5D). (2011). Factors associated with short-course antiretroviral prophylaxis (dual therapy) adherence for PMTCT in Nkangala district, South Africa. *Acta Paediatrica,* 100, 1253-1257.

122. Peltzer K, Mzolo T, Mbelle N,Tsoai L, Lewa N, Ncitakalo N. (2010). Dual protection, contraceptive use, HIV status and risk among a national sample of South African women. *Gender & Behavior,* 8, 1.

123. Peltzer K. (2012). Correlates of HIV infection among people visiting public HIV counseling and testing clinics in Mpumalanga, South Africa. *Afr Health Sci,* 12, 8-16.

[124. Peltzer K](http://www.ncbi.nlm.nih.gov/pubmed?term=%22Peltzer%20K%22%5BAuthor%5D), [Mosala T](http://www.ncbi.nlm.nih.gov/pubmed?term=%22Mosala%20T%22%5BAuthor%5D), [Shisana O](http://www.ncbi.nlm.nih.gov/pubmed?term=%22Shisana%20O%22%5BAuthor%5D), [Nqeteko A](http://www.ncbi.nlm.nih.gov/pubmed?term=%22Nqeteko%20A%22%5BAuthor%5D). (2006). Utilization of delivery services in the context of prevention of HIV from mother-to-child (PMTCT) in a rural community, South Africa. *Curationis,* 29, 54-61.

[125. Peltzer K](http://www.ncbi.nlm.nih.gov/pubmed?term=%22Peltzer%20K%22%5BAuthor%5D), [Mosala T](http://www.ncbi.nlm.nih.gov/pubmed?term=%22Mosala%20T%22%5BAuthor%5D), [Shisana O](http://www.ncbi.nlm.nih.gov/pubmed?term=%22Shisana%20O%22%5BAuthor%5D), [Nqueko A](http://www.ncbi.nlm.nih.gov/pubmed?term=%22Nqueko%20A%22%5BAuthor%5D), [Mngqundaniso N](http://www.ncbi.nlm.nih.gov/pubmed?term=%22Mngqundaniso%20N%22%5BAuthor%5D). (2007). Barriers to prevention of HIV transmission from mother to child (PMTCT) in a resource poor setting in the Eastern Cape, South Africa. *African Journal of Reproductive Health,* 11, 57-66.

126. Peltzer, K. & Mlambo, G. (2010a). Factors determining HIV viral testing of infants in the context of mother to child transmission. *Acta Paediatrica*, 99, 590-6.

127. Peltzer K & Mlambo G. (2013). Sexual HIV risk behaviour and associated factors among pregnant women in Mpumalanga, South Africa BMC Pregnancy and Childbirth 2013, 13:57

128. Peltzer, K. (2009). Utilization and practice of traditional/complementary/alternative medicine (TM/CAM) in South Africa. *African Journal of Traditional Complementary and Alternative Medicines*, 6, 175 – 185.

129. Peltzer, K., Chao, L.-W. & Dana, P. (2009). Family planning among HIV positive and negative Prevention of Mother to Child (PMTCT) clients in a resource poor setting in South Africa. *AIDS and Behavior*, 13, 973-9.

130. Peltzer, K., Mlambo, G., & Phaweni, K. (2010b). Factors determining prenatal HIV testing for prevention of mother to child transmission of HIV in Mpumalanga, South Africa. *AIDS and Behavior,* 14, 1115-1123.

131. Peltzer, K. & Shikwane, E. (2011) Prevalence of postnatal depression and associated factors among HIV positive women in primary care in Nkangala district, South Africa. *The Southern African Journal of HIV Medicine*, 12(4), 24-28.

132. Peltzer, K., Mlambo, G., Matseke, G., Shikwane, E., Louw, J. (2011). PMTCT comprehensive community intervention package including male involvement, infant follow-up, peer support, partner violence and infant feeding. *Nelspruit: Department of Health*.

133. Peltzer, K., Mlambo, M., Phaswana-Mafuya, N., & Ladzani, R. (2010c). Determinants of adherence to a single-dose nevirapine regimen for the prevention of mother-to-child HIV transmission in Gert Sibande district in South Africa. *Acta Paediatrica*, 99, 699-704.

134. Peltzer, K., Mosala, T., Dana, P., & Fomundam, H. (2008). Follow-up survey of women who have undergone Prevention of Mother to Child Transmission (PMTCT) in a resource poor setting in South Africa. *Journal of the Association of Nurses in AIDS Care*, 19, 450-460.

135. Peltzer, K., Phaswana-Mafuya, N., & Ladzani, R. (2010d). Implementation of the national programme for prevention of mother-to-child transmission of HIV: A rapid assessment in Cacadu district, South Africa. *African Journal of AIDS Research*, 9, 95-106.

136. Peltzer, K., Ramlagan, S., Jones, D., Weiss, S.M., Fomundam, H., & Chanetsa, L. (2012). Efficacy of a lay health worker led group antiretroviral medication adherence training among non-adherent HIV-positive patients in KwaZulu-Natal, South Africa: results from a randomized trial. *SAHARA J: Journal of Social Aspects of HIV/AIDS*, 9, 218-226.

137. Peltzer K, Sikwane E, Majaja M (2011). Factors associated with short-course antiretroviral prophylaxis (dual therapy) adherence for PMTCT in Nkangala district, South Africa*. Acta Pædiatrica* DOI:10.1111/j.1651-2227.2011.02253.x

138. Peltzer, K., Tabane, C., Matseke, G. & Simbayi, L. (2009) Lay counsellor-based risk reduction intervention with HIV negative diagnosed patients at HIV counselling and testing sites in a rural South African setting. Journal of Psychology in Africa, 19(4), 541-548.

139. Peltzer, K., Tabane, C., Matseke, G. & Simbayi, L. (2010) Lay counsellor-based risk reduction intervention with HIV positive diagnosed patients at public HIV counselling and testing sites in Mpumalanga, South Africa. Evaluation and Progam Planning, 33, 379-385.

140. Rispel, L.C., Peltzer, K., Phaswana-Mafuya, N., Metcalf, C.A. & Treger, L. (2009). Assessing missed opportunities for the prevention of mother-to-child HIV transmission (PMTCT) in the Kouga Local Service Area (LSA), Eastern Cape. *South African Medical Journal*, 99, 174-179.

141. Rollins N, Coovadia H, Bland R, Coutsoudis A, Bennish M, Patel D, Newell M, (2007). Pregnancy Outcomes in HIV-Infected and Uninfected Women in Rural and Urban South Africa. JAIDS, 44: 321-328.

142. Rosenberg NE, Pettifor AE, De Bruyn G, Westreich D, Delany-Moretlwe S, Behets F, Maman S, Coetzee D, Kamupira M, Miller WC. (2012). HIV Testing and Counseling Leads to Immediate Consistent Condom Use among South African Stable HIV-discordant Couples. *J Acquir Immune Defic Syndr*. [Epub ahead of print]

143. Rujumba J, Neema S, Byamugisha R, Tylleskär T, Tumwine JK, Heggenhougen HK. (2012). "Telling my husband I have HIV is too heavy to come out of my mouth": pregnant women's disclosure experiences and support needs following antenatal HIV testing in eastern Uganda. *J Int AIDS Soc*, 15, 17429.

[144. Russell BS](http://www.ncbi.nlm.nih.gov/pubmed?term=Russell%20BS%5BAuthor%5D&cauthor=true&cauthor_uid=23233038), [Eaton LA](http://www.ncbi.nlm.nih.gov/pubmed?term=Eaton%20LA%5BAuthor%5D&cauthor=true&cauthor_uid=23233038), [Petersen-Williams P](http://www.ncbi.nlm.nih.gov/pubmed?term=Petersen-Williams%20P%5BAuthor%5D&cauthor=true&cauthor_uid=23233038). (2012)Intersecting Epidemics Among Pregnant Women: Alcohol Use, Interpersonal Violence, and HIV Infection in South Africa. [Curr HIV/AIDS Rep.](http://www.ncbi.nlm.nih.gov/pubmed/23233038) 2012 Dec 12. [Epub ahead of print]

145. Rutenberg N, Baek C, Kalibala S, Rosen J. (2003). Evaluation of United Nations-supported pilot projects for the prevention of mother-to-child transmission of HIV. Overview of findings. Available at: <http://www.popcouncil.com/pdfs/horizons/pmtctunicefevalovrvw.pdf>.

146. Schwartz S, Taha TE, Venter WD, Mehta S, Rees H, Black V. (2012). Efavirenz Conceptions and Regimen Management in a Prospective Cohort of Women on Antiretroviral Therapy. *Infect Dis Obstet Gynecol*, 2012, 723096.

147. Schwartz SR, Mehta SH, Taha TE, Rees HV, Venter F, Black V. (2012). High Pregnancy Intentions and Missed opportunities for Provider communication about fertility in a South African cohort of HIV positive women on antiretroviral therapy. *AIDS & Behavior*, 16, 69-78.

148. Sengayi M, Dwane N, Marinda E, Sipambo N, Fairlie L, Moultrie H. (2013). Predictors of loss to follow-up among children in the first and second years of antiretroviral treatment in Johannesburg, South Africa. [*Glob Health Action, 24*:1-12.](http://www.globalhealthaction.net/index.php/gha/article/view/19305/html)

[149. Sethosa E](http://www.ncbi.nlm.nih.gov/pubmed?term=%22Sethosa%20E%22%5BAuthor%5D), [Peltzer K](http://www.ncbi.nlm.nih.gov/pubmed?term=%22Peltzer%20K%22%5BAuthor%5D). (2005). Evaluation of HIV counselling and testing, self-disclosure, social support and sexual behaviour change among a rural sample of HIV reactive patients in South Africa. [*Curationis*](http://www.ncbi.nlm.nih.gov/pubmed?term=sethosa%20e), 28, 29-41.

150. Seutlwadi L, Peltzer K. (2012). The use of dual or two methods for pregnancy and HIV prevention amongst 18-24-year-olds in a cross-sectional study conducted in South Africa. *Contraception*. [Epub ahead of print]

151. Shamu S, Abrahams N, Temmerman M, Musekiwa A, Zarowsky C. (2011). A systematic review of African studies on intimate partner violence against pregnant women: prevalence and risk factors. *PLoS One*, 6, e17591.

152. Shamu S, Abrahams N, Temmerman M, Shefer T, Zarowsky C. (2012). "That Pregnancy Can Bring Noise into the Family": Exploring Intimate Partner Sexual Violence during Pregnancy in the Context of HIV in Zimbabwe. *PLoS One*, 7(8), e43148.

153. Sherr L, Croome N. (2012). Involving fathers in prevention of mother to child transmission- what the evidence suggests. *J Int AIDS Soc*, 15 suppl 2, 17378.

154. Shisana, O., Simbayi, L.C., Rehle, T., Zungu, N.P., Zuma, K., Ngogo, N.,& … SABSSM III Implementation Team. (2010). South African National HIV Prevalence, HIV Incidence, Behaviour and Communication Survey, 2008: The health of our children. Cape Town: Human Sciences Research Council Press.

155. Simbayi LC, Kalichman SC, Strebel A, Cloete A, Henda N, Mqeketo A. (2007). Disclosure of HIV status to sex partners and sexual risk behaviours among HIV-positive men and women, Cape Town, South Africa. *Sexually Transmitted Infections,* 83, 29-34.

156. Sofolahan YA, Airhihenbuwa CO. Cultural Expectations and Reproductive Desires: Experiences of South African Women Living With HIV/AIDS (WLHA). (2013). *Health Care Women Int, 34*: 263-80.

157. Starace, F., Massa, A., Amico, K.R., & Fisher, J.D. (2006). Adherence to antiretroviral therapy: An empirical test of the information-motivation-behavioral skills model. *Health Psychology*, 25, 153-162.

158. Strauss, M. (1979). Measuring Intrafamily Conflict and Violence: The Conflict Scales. *Journal of Marriage and the Family,* 4*,* 75-88.

159. Straus MA (2004). Cross-cultural reliability and validity of the Revised Conflict Tactics Scales: a study of university student dating couples in 17 nations. *Cross-Cult Res*, *38*:407–432.

160. Straus MA (2008). Dominance and symmetry in partner violence by male and female university students in 32 nations. *Children Youth Serv Rev*, 30, 252–275.

161. Stringer EM, Chi BH, Namwinga C, Creek TL, Ekouevi DK, CoetzeeD, Stringer JSA. (2008). Monitoring effectiveness of programs to prevent mother-to-child transmission in lower-income countries. *Bulletin of the World Health Organization*, 86, 1-80.

162. Suthar AB, Hoos D, Beqiri A, Lorenz-Dehne K, McClure C, Duncombe C. (2013). Integrating antiretroviral therapy into antenatal care and maternal and child health settings: a systematic review and meta-analysis. *Bull World Health Organ, 91*: 46-56.

163. Tatagan A, Mouhari-Toure A, Saka B, Akakpo AS, Kombate D, Tchama R, Singo A, Mpélé K, Pitche P. (2011). Knowledge, attitudes and practices about prevention of mother to child transmission of HIV (PMTCT) among pregnant women in antenatal clinic at 2010 in Togo. *Medecine Tropicale*, 71, 472-476.

164. Theuring, S., Mbezi, P., Luvanda, H., Jordan-Harder, B., Kunz, A., & Harms G. (2009). [Male involvement in PMTCT services in Mbeya Region, Tanzania.](http://www.ncbi.nlm.nih.gov/pubmed/19308720?itool=EntrezSystem2.PEntrez.Pubmed.Pubmed_ResultsPanel.Pubmed_RVDocSum&ordinalpos=10) *AIDS & Behavior*, 13, 92-102.

[165. Tabana H](http://www.ncbi.nlm.nih.gov/pubmed?term=Tabana%20H%5BAuthor%5D&cauthor=true&cauthor_uid=22458410), [Doherty T](http://www.ncbi.nlm.nih.gov/pubmed?term=Doherty%20T%5BAuthor%5D&cauthor=true&cauthor_uid=22458410), [Swanevelder S](http://www.ncbi.nlm.nih.gov/pubmed?term=Swanevelder%20S%5BAuthor%5D&cauthor=true&cauthor_uid=22458410), [Lombard C](http://www.ncbi.nlm.nih.gov/pubmed?term=Lombard%20C%5BAuthor%5D&cauthor=true&cauthor_uid=22458410), [Jackson D](http://www.ncbi.nlm.nih.gov/pubmed?term=Jackson%20D%5BAuthor%5D&cauthor=true&cauthor_uid=22458410), [Zembe W](http://www.ncbi.nlm.nih.gov/pubmed?term=Zembe%20W%5BAuthor%5D&cauthor=true&cauthor_uid=22458410), [Naik R](http://www.ncbi.nlm.nih.gov/pubmed?term=Naik%20R%5BAuthor%5D&cauthor=true&cauthor_uid=22458410). (2012). Knowledge of HIV status prior to a community HIV counseling and testing intervention in a rural district of south Africa: results of a community based survey. [*BMC Infect Dis*.](http://www.ncbi.nlm.nih.gov/pubmed/22458410) 2012 Mar 29;12:73. doi: 10.1186/1471-2334-12-73.

166. Tanser F, Bärnighausen T, Grapsa E, Zaidi J, Newell M (2013). High Coverage of ART Associated with Decline in Risk of HIV Acquisition in Rural KwaZulu-Natal, South Africa. *Science, 339:* 996-970.

167. Taulo F, Berry M, Tsui A, Makanani B, Kafulafula G, Li Q, Nkhoma C, Kumwenda JJ, Kumwenda N, Taha TE. (2009). [Fertility intentions of HIV-1 infected and uninfected women in Malawi: a longitudinal study.](http://www.ncbi.nlm.nih.gov/pubmed/19308718) AIDS Behav. 2009 Jun;13 Suppl 1:20-7. doi: 10.1007/s10461-009-9547-9. Epub 2009 Mar 24.

168. Torpey K, Mandala J, Kasonde P, Bryan-Mofya G, Bweupe M, Mukundu J, Zimba C, Mwale C, Lumano H, Welsh M. (2012). Analysis of HIV Early Infant Diagnosis Data to Estimate Rates of Perinatal HIV Transmission in Zambia. PLoS One, 7(8), e42859.

[169. Horwood C](http://www.ncbi.nlm.nih.gov/pubmed?term=Horwood%20C%5BAuthor%5D&cauthor=true&cauthor_uid=20561313), [Haskins L](http://www.ncbi.nlm.nih.gov/pubmed?term=Haskins%20L%5BAuthor%5D&cauthor=true&cauthor_uid=20561313), [Vermaak K](http://www.ncbi.nlm.nih.gov/pubmed?term=Vermaak%20K%5BAuthor%5D&cauthor=true&cauthor_uid=20561313), [Phakathi S](http://www.ncbi.nlm.nih.gov/pubmed?term=Phakathi%20S%5BAuthor%5D&cauthor=true&cauthor_uid=20561313), [Subbaye R](http://www.ncbi.nlm.nih.gov/pubmed?term=Subbaye%20R%5BAuthor%5D&cauthor=true&cauthor_uid=20561313), [Doherty T](http://www.ncbi.nlm.nih.gov/pubmed?term=Doherty%20T%5BAuthor%5D&cauthor=true&cauthor_uid=20561313). (2010). Prevention of mother to child transmission of HIV (PMTCT) programme in KwaZulu-Natal, South Africa: an evaluation of PMTCT implementation and integration into routine maternal, child and women's health services. *Trop Med Int Health*, 15:992-9.

170. Tumwesigye NM, Wanyenze RK, Greenfield TK. (2012). Intoxication before last sexual intercourse and HIV risk behavior among men and women in Uganda: Evidence from a nationwide survey. *Int J Alcohol Drug Res,* 1, 17.

171. Turan JM, Bukusi EA, Onono M, Holzemer WL, Miller S, Cohen CR. (2011). HIV/AIDS Stigma and Refusal of HIV Testing Among Pregnant Women in Rural Kenya: Results from the MAMAS Study. *AIDS Behav*, 15, 1111-1120.

172. Turan JM, Hatcher AH, Medema-Wijnveen J, Onono M, Miller S, Bukusi EA, Turan B, Cohen CR. (2012). The Role of HIV- Related Stigma in Utilization of Skilled Childbirth Services in Rural Kenya: A Prospective Mixed-Methods Study. *PLoS Med,* 9(8), e1001295.

173. Ukpe IS, Blitz J, Hugo J, Theledi M. (2009). The infant-feeding practices of mothers enrolled in the prevention of mother-to-child transmission of HIV programme at a primary health care clinic in the Mpumalanga province, South Africa. *South African Family Practice,* 51, 337-339.

174. UNAIDS (2012). UNAIDS World AIDS Day Report. http://www.unaids.org/en/aboutunaids/

175. Van de Perre P, Rubbo PA, Viljoen J, Nagot N, Tylleskär T, Lepage P, Vendrell JP, Tuaillon E. (2012). HIV-1 Reservoirs in Breast Milk and Challenges to Elimination of Breast-Feeding Transmission of HIV-1. *Sci Transl Med*, 4(143), 143sr3.

[176. Uwimana J](http://www.ncbi.nlm.nih.gov/pubmed?term=Uwimana%20J%5BAuthor%5D&cauthor=true&cauthor_uid=22853044), [Zarowsky C](http://www.ncbi.nlm.nih.gov/pubmed?term=Zarowsky%20C%5BAuthor%5D&cauthor=true&cauthor_uid=22853044), [Hausler H](http://www.ncbi.nlm.nih.gov/pubmed?term=Hausler%20H%5BAuthor%5D&cauthor=true&cauthor_uid=22853044), [Jackson D](http://www.ncbi.nlm.nih.gov/pubmed?term=Jackson%20D%5BAuthor%5D&cauthor=true&cauthor_uid=22853044). (2012). Engagement of non-government organisations and community care workers in collaborative TB/HIV activities including prevention of mother to child transmission in South Africa: opportunities and challenges. [*BMC Health Serv Res,*](http://www.ncbi.nlm.nih.gov/pubmed/22853044) *12*:233. doi: 10.1186/1472-6963-12-233.

177.

[178. Uwimana J](http://www.ncbi.nlm.nih.gov/pubmed?term=Uwimana%20J%5BAuthor%5D&cauthor=true&cauthor_uid=22296235), [Zarowsky C](http://www.ncbi.nlm.nih.gov/pubmed?term=Zarowsky%20C%5BAuthor%5D&cauthor=true&cauthor_uid=22296235), [Hausler H](http://www.ncbi.nlm.nih.gov/pubmed?term=Hausler%20H%5BAuthor%5D&cauthor=true&cauthor_uid=22296235), [Jackson D](http://www.ncbi.nlm.nih.gov/pubmed?term=Jackson%20D%5BAuthor%5D&cauthor=true&cauthor_uid=22296235). (2012).Training community care workers to provide comprehensive TB/HIV/PMTCT integrated care in KwaZulu-Natal: lessons learnt. [*Trop Med Int Health,*](http://www.ncbi.nlm.nih.gov/pubmed/22296235)*17*:488-96.

179. Vernazza PL, Graf I, Sonnenberg-Schwan U, Geit M, Meurer A. (2011). Pre-prophylaxis and timed intercourse for HIV-discordant couples willing to conceive a child. *AIDS*, 25, 433-45.

180. Villar-Loubet, O.M., Bruscantini, L., Shikwane, M.E., Weiss, S., Peltzer, K., Jones, D.L. (2012). HIV disclosure, sexual negotiation and male involvement in prevention-of-mother-to-child-transmission in South Africa. Culture, Health & Sexuality: An International Journal for Research, Intervention and Care. [Epub ahead of print]

181. Villar-Loubet OM, Cook R, Chakhtoura N, Peltzer K, Weiss SM, Shikwane ME, Jones DL. (2012). HIV Knowledge and Sexual Risk Behavior Among Pregnant Couples in South Africa: The PartnerPlus Project. AIDS & Behavior. [Epub ahead of print]

182. Visser M, Neufeld S, de Villiers A, Makin JD, Forsyth B. (2008). To tell or not to tell: South African women's disclosure of HIV status during pregnancy. *AIDS Care: Psychological and Sociomedical Aspects of AIDS/HIV*, 20, 1138-1145.

183. Vu L, Andrinopoulos K, Mathews C, Chopra M, Kendall C, Eisele TP. (2012). Disclosure of HIV Status to Sex Partners Among HIV-Infected Men and Women in Cape Town, South Africa. *AIDS & Behavior*, 16, 132-138.

184. Walker AS, Prendergast AJ, Mugyenyi P, Munderi P, Hakim J, Kekitiinwa A, Katabira E, Gilks CF, Kityo C, Nahirya-Ntege P, Nathoo K, Gibb DM; on behalf of the DART and ARROW trial teams. Mortality in the year following antiretroviral therapy initiation in HIV-infected adults and children in Uganda and Zimbabwe. *Clin Infect Dis*, 55, 1707-1718.

185. Wall KM, Kilembe W, Nizam A, Vwalika C, Kautzman M, Chomba E, Tichacek A, Sardar G, Casanova D, Henderson F, Mulenga J, Kleinbaum D, Allen S. (2012). Promotion of couples' voluntary HIV counselling and testing in Lusaka, Zambia by influence network leaders and agents. *BMJ Open*, 2(5), e001171.

186. Wall KM, Vwalika B, Haddad L, Khu NH, Vwalika C, Kilembe W, Chomba E, Stephenson R, Kleinbaum D, Nizam A, Brill I, Tichacek A, Allen S. (2012). Impact of long-term contraceptive promotion on incident pregnancy: a randomized controlled trial among HIV positive couples in Lusaka, Zambia. *J Acquir Immune Defic Syndr*. [Epub ahead of print]

187. Wanyenze RK, Wagner GJ, Tumwesigye NM, Nannyonga M, Wabwire- Mangen F, Kamya MR Fertility and contraceptive decision-making and support for HIV infected individuals: client and provider experiences and perceptions at two HIV clinics in Uganda. (2013). *BMC Public Health*,*13*: 98. [Epub ahead of print] PMID 23438041

188. Watson-Jones D, Balira R, Ross DA, Weiss HA, Mabey D. (2012). Missed Opportunities: Poor Linkage into Ongoing Care for HIV-Positive Pregnant Women in Mwanza, Tanzania. *PLoS One*, 7(7), e40091.

189. Weiss, S.M., Jones, DL, Lopez, M, Villar-Loubet, O, Chitalu, N. (2011). The Many Faces of Translational Research: A Tale of Two Studies. *Translational Behavioral Medicine,* 1, 327-330.

190. Westreich D, Maskew M, Rubel D, MacDonald P, Jaffray I, Majuba P. (2012). Incidence of Pregnancy after Initiation of Antiretroviral Therapy in South Africa: A Retrospective Clinical Cohort Analysis. *Infect Dis Obstet Gynecol*, 2012, 917059.

191. Wettstein C, Mugglin C, Egger M, Blaser N, Vizcaya LS, Estill J, Bender N, Davies MA, Wandeler G, Keiser O; for the IeDEA Southern Africa Collaboration. (2012). Missed opportunities to prevent mother-to-child-transmission: systematic review and meta-analysis. *AIDS*, 26, 2361-2373.

192. WHO & The Partnership for Maternal, Newborn and Child Health (WHO PANCH). (2006). Opportunities for Africa’s Newborns: Practical data, policy and programmatic support for newborn care in Africa. Joy Lawn and Kate Kerber, eds. PMNCH, Cape Town, 2006. Available at: <http://www.who.int/pmnch/media/publications/oanexecsum.pdf>

193. WHO & The Partnership for Maternal, Newborn and Child Health. (2006). Warren, C, Daly, P, Toure, L, Mongi, P. Opportunities for Africa’s newborns. Chapter 4. Postnatal care. Eds. Joy Lawn, Save the Children, Kate Kerber, eds. PMNCH, Cape Town, 2006. Available at: <http://www.who.int/pmnch/media/publications/aonsectionIII_4.pdf>

194. WHO, UNICEF, The Integrated Task Team on Prevention of HIV. (2007). Guidance on global scale up of the prevention of mother to child transmission of HIV. Towards universal access for women, infants and young children and eliminating HIV and AIDS among children*.* Geneva.

195. WHO. (2001). Breastfeeding and replacement feeding practices in the context of mother-to-child transmission of HIV: An assessment tool for research*.* Geneva.

196. WHO. (2002). Strategic Approaches to the Prevention of HIV Infection in Infants. Report of a WHO Meeting, Morges, Switzerland, 20-22nd, Mar. Available at: <http://www.who.int/hiv/pub/mtct/en/>

197. WHO. (2010). WHO Technical Consultation on Postpartum and Postnatal Care. Available at: http://whqlibdoc.who.int/hq/2010/WHO_MPS_10.03_eng.pdf.

198. WHO. (2012). Global Health Observatory. Available at: <http://www.who.int/gho/en/>

199. WHO. (2012). Global HIV/AIDS response – Epidemic update and health sector progress towards Universal Access - Progress report summary 2011. Available at: <http://www.who.int/hiv/pub/progress_report2011/summary_en.pdf>

200. WHO/UNFPA. (2006). Sexual and reproductive health of women living with HIV/AIDS: guidelines on care, treatment and support for women living with HIV/AIDS and their children in resource-constrained settings. Available at: http://www.who.int/hiv/pub/guidelines/sexualreproductivehealth.pdf.

201. WHO. (2010). Strategic vision 2010-2015: Preventing mother-to-child transmission of HIV to reach the UNGASS and millennium development goals. Available at: http://www.who.int/hiv/pub/mtct/strategic_vision/en/index.html.
